# Improving enhanced recovery after surgery (ERAS): ERAS APPtimize study protocol, a randomized controlled trial investigating the effect of a patient-centred mobile application on patient participation in colorectal surgery

**DOI:** 10.1186/s12893-019-0588-3

**Published:** 2019-09-02

**Authors:** A. Rauwerdink, M. Jansen, C. A. J. M. de Borgie, W. A. Bemelman, F. Daams, M. P. Schijven, C. J. Buskens

**Affiliations:** 1 Department of surgery, Amsterdam Gastroenterology and Metabolism, Amsterdam UMC, University of Amsterdam, Meibergdreef 9, Amsterdam, Netherlands; 2 Clinical Research Unit, Amsterdam UMC, University of Amsterdam, Amsterdam, Netherlands; 3 Department of surgery, Amsterdam Gastroenterology and Metabolism, Amsterdam UMC, Vrije Universiteit Amsterdam, Amsterdam, Netherlands

**Keywords:** ERAS, Colorectal surgery, eHealth, mHealth, Mobile application

## Abstract

**Background:**

Perioperative care in colorectal surgery is systematically defined in the Enhanced Recovery After Surgery (ERAS) protocol. The ERAS protocol improves perioperative care in a multimodal way to enhance early and safe release from the hospital. Adequate compliance to the elements of the ERAS protocol is multifactorial. There are still opportunities to improve compliance of the protocol by actively involving the patient. The main objective of this study is to investigate whether compliance of selected items in the ERAS protocol can be improved through actively involving patients in the ERAS care pathway through the use of a patient-centred mobile application.

**Methods:**

A multicentre randomized controlled trial will be conducted. Patients undergoing elective colorectal surgery, who are 18 years or older and in possession of an eligible smartphone, will be included. Patients assigned to the intervention group will install a patient-centred mobile application to be guided through the ERAS care pathway. Patients in the control group will receive care as usual. Both groups will wear an activity tracker. The primary outcome is overall compliance to selected active elements of the ERAS protocol, as registered by the patient. Secondary outcomes include Patient Reported Outcome Measures (PROMs) such as health-related quality of life, physical activity, and patient satisfaction of received care. Care-related outcomes, such as length of hospital stay, number of complications, re-intervention, and readmission rates, will also be assessed.

**Results:**

The enrolment of patients will start in the second quarter of 2019. Data collection had not begun by the time this protocol was submitted.

**Conclusion:**

We hypothesize that by providing patients with a patient-centred mobile application, compliance to the active elements of ERAS protocol can be improved, resulting in an increased health-related quality of life, physical activity, and patient satisfaction.

**Trial registration:**

Netherlands Trial Register, NTR7314, prospectively registered on the 9th of November 2017 (http://www.trialregister.nl).

**Electronic supplementary material:**

The online version of this article (10.1186/s12893-019-0588-3) contains supplementary material, which is available to authorized users.

## Background

The Enhanced Recovery After Surgery (ERAS) Society was formed in 2001 by a group of European surgeons to optimize clinical outcomes of patients undergoing surgery. Initially the ERAS Study Group published a consensus document with a scientific, evidence-based approach about the perioperative care for colonic resections (2005) and colorectal surgery (2009) [1]. Compared to routine care in elective colonic surgery, application of the ERAS protocol proved to reduce surgical stress, resulting in a better postoperative physiological status of the patient and improved mobilization short-term after surgery [2–6]. These outcomes contribute to faster postoperative recovery, shorter length of stay in hospital (LOS), and reduced rates of morbidity [7–9]. Nowadays, the ERAS protocol consists of 24 core elements (Table 1), and in order to successfully implement these 24 elements, a multidisciplinary team of anaesthetists, surgeons, nurses, physiotherapist and dieticians is essential. Local ERAS protocols, based on the ERAS guideline, can vary depending to the performing medical centre. The ERAS protocol elements are divided into preadmission, preoperative, intraoperative and postoperative phases, which, in their synergy, improve recovery after surgery [10]. An element is considered ‘active’ when some or full contribution of the patient is required. Elements are ‘passive’ when they do not directly depend on or require patient’s actions (Table 1) [11].

**Table 1:** ERAS society guideline elements for colonic resections

Element	Responsible professional	Involvement patient
Preadmission		
1 Cessation of smoking and excessive intake of alcohol	Surgeon, patient	Active
2 Preoperative nutritional screening and, as needed, assessment and nutritional support	Surgeon	Active/Passive
3 Medical optimization of chronic disease	Anaesthetist	Passive
Preoperative		
1 Structured preoperative information and engagement of the patient and relatives or caretakers	Nurse	Passive
2 Preoperative carbohydrate treatment	Nurse	Active/Passive
3 Preoperative prophylaxis against thrombosis	Surgeon	Active/Passive
4 Preoperative prophylaxis against infection	Anaesthetist	Passive
5 Prophylaxis against nausea and vomiting	Anaesthetist	Passive
Intraoperative		
1 Minimal invasive surgical techniques	Surgery	Passive
2 Standardized anesthesia, avoiding long-acting opioids	Anaesthetist	Passive
3 Maintaining fluid balance to avoid over- or under hydration, administer vasopressors to support blood pressure control	Anaesthetist	Passive
4 Epidural anesthesia for open surgery	Anaesthetist	Passive
5 Restrictive use of surgical site drains	Surgeon	Passive
6 Removal of nasogastric tubes before reversal of anesthesia	Anaesthetist	Passive
7 Control of body temperature using warm air flow blankets and warmed intravenous infusions	Anaesthetist	Passive
Postoperative		
1 Early mobilization (day of surgery)	Patient	Active
2 Early intake of oral fluids and solids (offered the day of surgery)	Patient	Active
3 Early removal of urinary catheters and intravenous fluids (morning after surgery)	Nurse	Active/Passive
4 Use of chewing gums and laxatives and peripheral opioid-blocking agents (when using opioids)	Patient	Active
5 Intake of protein and energy-rich nutritional supplements	Patient	Active
6 Multimodal approach to opioid-sparing pain control	Anaesthetist	Passive
7 Multimodal approach to control of nausea and vomiting	Anaesthetist	Passive
8 Prepare for early discharge	Nurse, patient	Active/Passive
9 Audit of outcomes and process in a multi-professional, multidisciplinary team on a regular basis	Whole team	Passive

In addition to regular use of the protocol, the ERAS Society recommends conducting a systematic audit to gather insights into clinical- and care-related outcomes, such as LOS, readmission, and postoperative complications, and to measure protocol compliance [9]. Studies investigating ERAS protocol compliance demonstrate that higher compliance rates are significantly associated with improved clinical outcomes such as shorter LOS, fewer postoperative complications, reduced 30-day morbidity, and reduced readmission rates [12–15]. Messenger et al. analysed the protocol compliance of 21 individual ERAS elements in a systematic review. The pooled results of 12 studies showed a 69, 72, and 53% adherence to the protocol within the pre-, peri- and postoperative phases [16]. ERAS protocol deviation is considered to be most critical in the postoperative phase, when mobilization and resumption of oral intake should be stimulated in order to not delay hospital discharge and to minimize postoperative complications. Improving postoperative protocol compliance is challenging but there is certainly room for improvement [17], especially since the majority of the postoperative elements are considered ‘active’ and, therefore, depend on patients’ actions directly.

With regards to improving patient participation in the ERAS care pathway, innovative technologies, such as mobile applications and wireless monitoring, could have great potential [18, 19]. Use of these eHealth solutions by patients can be educational, engaging, and stimulating. It might also enhance empowerment and let patients feel more in control of their own health [20]. Cook et al. used a wireless accelerometer to monitor patients postoperatively and demonstrated a significant relationship between the number of steps taken in the early recovery period and LOS in an older cardiac surgery population [21]. A small study of Mundi et al. showed that using a smartphone application for education and engagement of patients prior to bariatric surgery could be beneficial [22].

By conducting this randomized controlled trial (RCT) we want to investigate whether a patient-centred mobile application can significantly improve compliance to the active elements of the ERAS protocol significantly by patients undergoing colorectal surgery.

## Methods

### Study setting

The ERAS APPtimize study is a multicentre RCT that will be conducted in the Amsterdam University Medical Centres (UMCs), locations AMC and VUMC, in the Netherlands. APPtimize is a blended word, combining ‘APP’ and ‘timize’ from ‘application’ and ‘optimization’. The SPIRIT (Standard Protocol Items: Recommendations for Interventional Trials) 2013 statement will be followed and the trial will be reported in accordance to the CONSORT-EHEALTH(Consolidated Standards of Reporting Trials of Electronic and Mobile Health Applications and online TeleHealth) checklist V1.6.2. A completed informed consent form is required to participate in this study. The ERAS APPtimize study will be conducted in line with the declaration of Helsinki. Approval of the local medical ethics committee for this study was obtained (registration number NL63874.018.17) and the study is registered at the Netherlands Trial Registry (NTR7314).

### Study population

The study population consists of patients scheduled to undergo colorectal surgery for either benign or malignant conditions. Patients must be 18 years or older and in possession of a smartphone operating iOS 9 (release date: September 16, 2015) and up or android 8.0 (release date: August 21, 2017) and up. Participants who meet one or more of the following criteria will not be considered for inclusion:

Exclusion criteria:

- Palliative surgery or surgery following neo-adjuvant radio- or chemotherapy
- Elective surgery for previously established complications (e.g. enteral fistula, presacral abscess) with the exception of colostomy correction
- Patients with a Karnofsky score ≤ 40
- Incompetence of understanding the Dutch language
- Visual impairment, unless well corrected with visual aids
- Physical or mental disabilities limiting the use of a mobile application
- When pre-operatively is estimated by the treating surgeon that adherence to the ERAS protocol postoperative is not feasible
- If expected LOS is 3 days or less after surgery
- Multiple organ resection

### Investigational intervention

#### Content development

A consensus meeting was organized with a multidisciplinary team of caregivers, representing both Amsterdam UMCs’ locations. Both locations practice almost all the elements from the 2012 ERAS society recommendations for colorectal surgery as part of their ‘care as usual’ except for ‘nutritional screening’ and ‘chewing gum as a laxative’. [9] Active ERAS elements, elements that depend on patient involvement, were included in the application. The multidisciplinary team of caregivers evaluated the ERAS elements for their eligibility to be included in the application. Although ‘nutritional screening’ was not part of current ‘care as usual’, the multidisciplinary team thought it would be useful to include this element in the application. Table 2 displays all elements that were included in the application. In ‘Additional file 1’, one can find the functional design of the application. The functional design illustrates the workflow through the application and the basic layout. After the first version of the application will be developed, ‘patient experts’ will be invited to test the application and review the content of the application.

**Table 2:** ERAS elements in the APPtimize application

Preadmission	
1. Cessation of smoking and excessive intake of alcohol	
2. Preoperative nutritional screening and, as needed, assessment and nutritional support	
Preoperative	
3. Preoperative carbohydrate treatment	
4. Preoperative prophylaxis against thrombosis	
Postoperative	
5. Early mobilization (day of surgery)	
6. Early intake of oral fluids and solids (offered the day of surgery)	
7. Early removal of urinary catheters and intravenous fluids (morning after surgery)	
8. Use of laxatives	
9. Intake of protein and energy-rich nutritional supplements	
10. Prepare for early discharge	

#### Technological development

The application is developed by a third party. The application will work on smartphones operating iOS 9 (release date: September 16, 2015) and up or android 8.0 (release date: August 21, 2017) and up. Applications for smartphone destined for diagnosis, prevention, monitoring, or relieve of diseases are considered medical devices [23]. The application used in the APPtimize study is developed specifically for patients having to undergo colorectal surgery and is therefore considered a medical device. Therefore, the application is submitted for CE-marking.

#### Activity tracker

We chose to use an activity tracker from the company Fitbit because it is deemed to provide the best accuracy results, as reflected upon in available literature [24, 25]. The selected Fitbit type is the Flex 2, which does not have a visual display on the bracelet. The intervention group receives feedback about their activity through the APPtimize application but the control group should not receive any feedback about their activity. Therefore, using an activity tracker without a visual display, is, to our belief, the most appropriate way to minimize information bias. The activity tracker will monitor physical activity, measured in steps per day, in both groups. Other information that is captured with the Flex 2 is not represented in the ERAS APPtimize application. Participants of both study groups have to wear the tracker continuously, from 7 days prior to surgery until 21 days after surgery. The battery of the activity tracker will last about 5 days, therefore patients will be instructed to charge their activity tracker during the night. As for the intervention group, the activity data will be stored in the database automatically via the mobile application. The participants in the control group are instructed to send the activity tracker to the coordinating researcher after the 4 weeks of use. The corresponding researcher will process the activity data of the control group to the database manually through the Fitbit application.

#### Usability testing

The usability of the app will be tested by a group of ‘patient experts’. Members of different patient associations with expertise in colorectal diseases and surgery were approached to test the application. Through multiple cognitive walkthrough sessions, the weaknesses of the application will be determined and adjustments can be made. Furthermore, the use of the applications will continuously be monitored in order to improve the application during and after completion of this trial.

### Control and intervention group

#### APPtimize study group

**Fig. 1:**
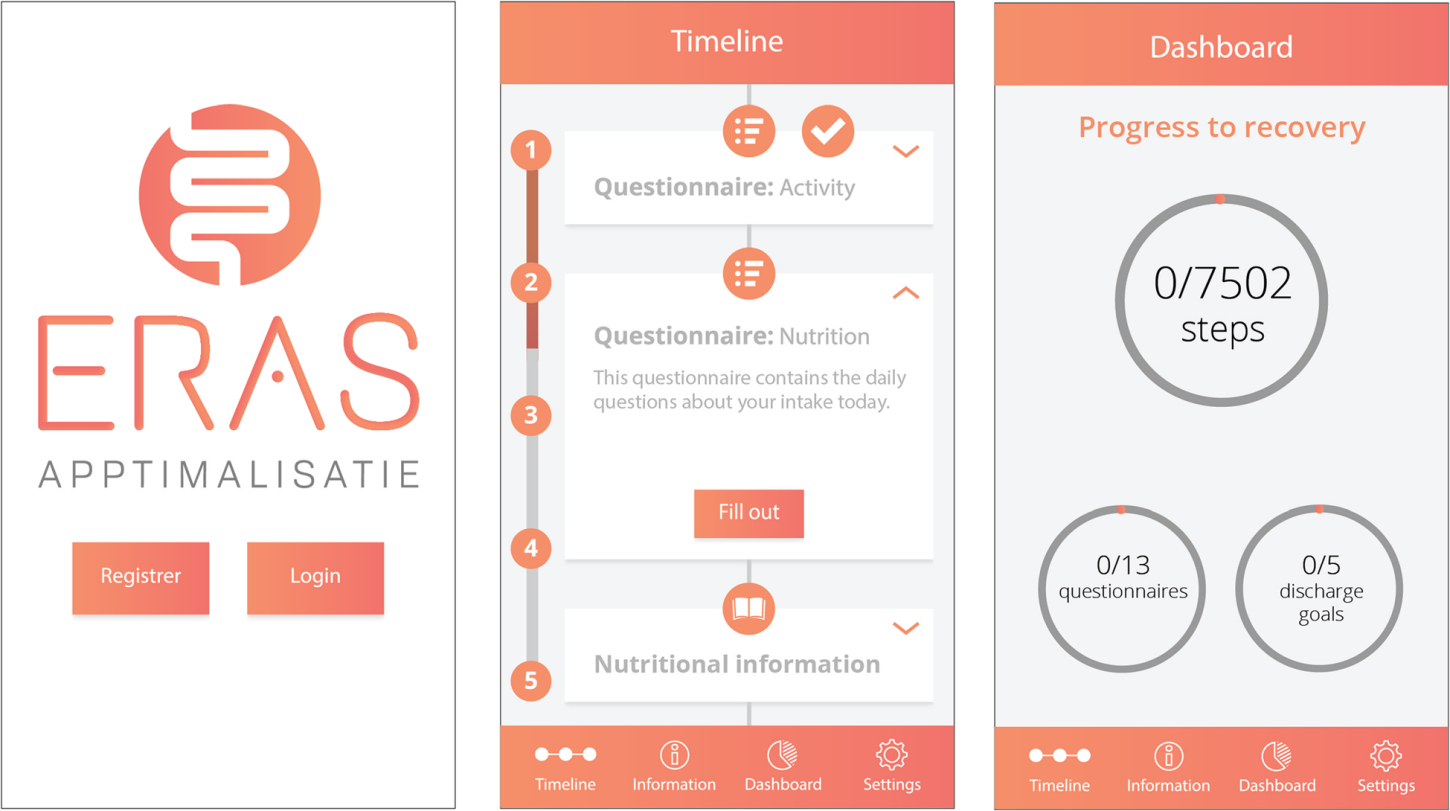
Screenshots APPtimize mobile application. NB Written permission to publish content relating to the APPtimize mobile application was obtained from the designer

Patients in the intervention group will receive instructions how to download the APPtimize application shortly after the operation is scheduled. The application will be used until 42 days postoperative. The selected ERAS elements (Table 2) have been translated into practical patient-tailored features that can be viewed in the application any time but will also be brought to the patient’s attention through push notifications (Table 3) at set times. The push notifications attempt to prompt the patient to undertake action towards an ERAS element that requires action at that specific moment. The main goals of the application are: to inform and educate the patient, to stimulate patient participation throughout the perioperative care pathway, and to monitor daily activity. All information provided by the application can be accessed at any moment but also once an ERAS element of the care pathway is considered completed. Figure 1 illustrates the layout of the application, with the third screenshot showing the application’s feature ‘dashboard’. This feature represents the completion of three subjects: 1. completion of the daily set activity goal 2. completion of the active ERAS elements, and 3. completion of self-registered questionnaires throughout the entire study.

**Table 3:** Push notifications

Preoperative day	Notification
−21	Information about nutrition
−20	Information about smoking
−16	Information about preparation before surgery
−8	Instructions to wear the activity tracker
−2	No alcohol 24 h before surgery
−1	Instructions to drink the pre-operative nutrition drink (4x)
0	Instructions to drink the pre-operative nutrition drink (1x)
Postoperative day	Notification
0	Instructions to wear the activity tracker
1,2,3,..,day of discharge	information of daily goals
1,2,3,..,day of discharge	Information on progress of daily activity
1,2,3,..,day of discharge	Check progress to discharge in the overview of ‘completion of goals’

To measure daily activity, the patient will be instructed to continuously wear the activity tracker, starting 7 days prior to hospital admission or as soon as possible after surgery is scheduled if this period is less than 7 days. The mean daily step count during this preoperative period will be used as a baseline reference value that will be used to calculate the individual postoperative daily step count goal. The postoperative daily step count goal is based on a daily increasing percentage of the mean preoperative daily step count (Table 4). The percentages are comparable to the usual activity instructions given by the local physiotherapist and generally recommended in literature [26]. Patients will receive instructions to wear the activity tracker until 21 days postoperative, since the biggest increase in activity is expected during this period [27].

**Table 4:** Daily step goal

	Days	Registration
Preadmission	7	Daily step count^a^
	Days	Daily step goal
Postoperative	0	50
	1	2,0% * preadmission step mean^b^
	2	6,7% * preadmission step mean^b^
	3–5	19,6% * preadmission step mean^b^
	6–8	28,9% * preadmission step mean^b^
	9–11	38,3% * preadmission step mean^b^
	12–14	47,7% * preadmission step mean^b^
	15–17	57,0% * preadmission step mean^b^
	18–20	66,5% * preadmission step mean^b^
	21	76,0% * preadmission step mean^b^

^a^days are valid measurements when activity tracker is worn ≥10 h

^b^Daily step mean = (Steps/day preadmission)/number of measured days

#### Control group

The control group will receive ‘care as usual’. However, to accurately compare pre- and postoperative activity between both groups, the control group will be instructed to also wear an activity tracker 7 days prior to hospital admission or as soon as possible after surgery is scheduled if this period is less than 7 days. The activity tracker will have to be worn until 21 days after surgery. No feedback is provided through the activity tracker to the control group patient, as it might influence the daily activity. To register the completion of the ERAS elements of the past day, patients of the control group will be instructed to fill out a checklist of completion of the ERAS elements in a diary once a day.

### Outcomes

The primary outcome is the overall average compliance to the selected active ERAS elements (Table 2). Since the application is patient-centred and focusses on empowering patients to get in control of their own health, the PROMs, such as health related quality of life and patient satisfaction, are considered important secondary outcome parameters. Other secondary outcomes include postoperative outcome parameters (e.g. complications), gastro-intestinal recovery (e.g. time to passage of stool), activity and pain measurements. Table 5 describes all study outcomes and how and when these will be measured.

**Table 5:** Time points and measurements

Enrolment		T0	T1	T2	T3	T4	T5	T6	T7	Registered in/with:
	Eligibility screen	x								
	Informed consent	x								
	Allocation	x								
Interventions		T0	T1	T2	T3	T4	T5	T6	T7	
Intervention group	Download mobile application	x								
	Wear activity tracker		O	O	O	O	O	O		
Control group	Wear activity tracker		O	O	O	O	O	O		
Data measurements		T0	T1	T2	T3	T4	T5	T6	T7	
Baseline characteristics	General characteristics	x								Sex, age, ASA, BMI, smoking, alcohol intake
	Disease related characteristics	x								Disease, operation
	eHealth literacy assessment	x								eHEALS
Protocol compliance *(as reported by patients)*	1. Cessation of smoking and excessive intake of alcohol	x								Y/N questions in app or with paper form
	2. Preoperative nutritional screening and, as needed, assessment and nutritional support	x								Y/N questions in app or with paper form
	3. Preoperative carbohydrate treatment			x						Y/N questions in app or with paper form
	4. Preoperative prophylaxis against thrombosis			x						Y/N questions in app or with paper form
	5. Early mobilization (day of surgery)				O	O				Y/N questions in app or with paper form
	6. Early intake of oral fluids and solids (offered the day of surgery)				O	O				Y/N questions in app or with paper form
	7. Early removal of urinary catheters and intravenous fluids (morning after surgery)					O				Y/N questions in app or with paper form
	8. Use of laxatives					O				Y/N questions in app or with paper form
	9. Intake of protein and energy-rich nutritional supplements					O				Y/N questions in app or with paper form
	10. Prepare for early discharge					O				Y/N questions in app or with paper form
Postoperative data ≤30 days *(retrospective collection)*	Length of hospital stay								x	EHR
	Overall morbidity ≤30 days								x	EHR
	Complications								x	Clavien-Dindo minor: I-II major: III-V
	Reoperations								x	EHR
	Readmission ≤30 days								x	EHR
	In-hospital mortality								x	EHR
Gastrointestinal	Tolerance of solid food				O					Days
	Absence of nausea				O					Days
	Passage of first flatus				O					Days
	Passage of first stool				O					Days
	Weight - preoperative			x						Weight at admission, KG
	Weight - at discharge				x					Weight at discharge, KG
Activity	Mean preoperative physical activity (steps/day)		O							Activity tracker, steps per day
	Postoperative physical activity (steps/day)				O	O	O			Steps per day
Pain	Perceived pain daily postoperative - discharge				O					NRS 1–10*
	Compliance with intake of (pain) medication				O					Y/N questions in app or with paper form
Self-registered questionnaires		T0	T1	T2	T3	T4	T5	T6	T7	
PROMS	General quality of life (WHOQol)	x				x	x		x	WHOQoL - BREF
	Multidimensional fatigue assessment	x						x	x	Multidimensional fatigue inventory
	Physical Activity (IPAQ)	x				x		x	x	IPAQ-short
	Disability	x							x	WHODAS2
	Patient satisfaction questionnaire								x	Self-developed patient satisfaction questionnaire

T0: Moment of randomization; T1: 1–7 days before stay in hospital; T2 hospital admission; T3: day of surgery; T4: 1–7 days after surgery; T5: 8–14 days after surgery; T6: 15 days – 21 days after surgery; T7: 22–42 days after surgery

x: the event/action is performed or assessed once in the indicated time slot. O: the event/action is performed or assessed continuously in the indicated time slot. *Numeric rating scale

### Recruitment

The coordinating researcher of the APPtimize trial will screen weekly the colorectal outpatients clinic lists per centre for eligible participants. The treating physician will be asked to get permission from the patients to be approached by the coordinating researcher. The treating physician hands out the Patient Information Form (PIF) when patients agree to be approached by the coordinating researcher. After permission to be approached and ‘informed consent for screening’ is obtained, the coordinating researcher will call the patient to explain the study and address any questions the patient may have after reading the PIF. After the telephone conversation, patients will be granted a reasonable amount of decision time - at least a minimum of 72 h - to decide whether they want to participate. Reasons of refusing participation will be registered and patients are asked permission for collection of postoperative outcome parameters. After obtaining written consent, participants will be randomized.

### Group allocation and blinding

After inclusion, participants are randomly assigned to the mobile application (intervention) group or standard care (control) group in a 1:1 ratio, using an internet randomization module with stratification for benign or malignant pathology and age (> 50 years and < 50 years). Random block sizes of 2, 4, and 6 will be used. The coordinating researcher will initiate the allocation sequence and following study enrolment.

Participants, professionals of the healthcare team, and outcome assessors will not be blinded to the treatment allocation. Patients will be instructed to not tell other patients in their ward if they were assigned to the intervention or control group.

### Data collection

Data from the intervention group will mostly be automatically collected and stored in the database. For instance, results from the self-reported questionnaires will be sent from the application to the database. Some data will be collected through the Electronic Health Record (EHR) by the coordinating researcher and entered in case report forms (CRFs). As for the control group, the data from the patient dairy, the self-registered questionnaires, and data from the activity tracker will be manually entered in the CRF. Trial findings will be stored in accordance with local data protection laws and handled in confidence. A data protection impact assessment has been part of the protocol.

### Sample size calculation

Compliance with the active ERAS elements is described in literature. The study population and data analyses of the study of Thorn et al. shares similarities with our proposed study [11]. Therefore, we used results from the study of Thorn et al. as references values to calculate the sample size. We assumed that the two ERAS elements ‘early mobilization’ and ‘adequate intake’ depended the most on participation of the patient and to which a mobile application that stimulates the patient to mobilize and to follow a normal diet, could contribute the most. The average compliance of these two elements is 57% [11]. We hypothesize that the overall average compliance percentage of the selected active elements in the APPtimize study group will increase to 62%. Although an increase of 5% might not seem clinically relevant, we expect that the some individual ERAS elements, such as early mobilization, even show a greater increase and therefore enhance clinical utility. Through a sample size calculation with 90% power, a 2-sided alpha of 0.05, and a standard deviation of 9, we estimated that 70 participants per study group are needed. A loss to follow-up of 10% was estimated. Therefore, the total target sample size is 156 participants ((2 × 70) / 0.9 = 156).

### Data analyses

Statistical analyses of any difference between the two study groups will be performed using SPSS for Windows version 25 or higher (SPSS Inc. Chicago, IL). Data will be analysed according to intention to treat protocol. If applicable, missing data will be imputed. Baseline characteristics will be summarized using descriptive statistics and compared between the intervention and control groups. Continuous data will be reported as mean and standard deviation in case of normal distribution and as median 95% confidence intervals in case of non-normal distribution. Normality of the data distribution will be analysed by visually inspecting the histograms and Kolmogorov-Smirnov test. Comparative analysis will be done using a two-sided t-test in case of normal distribution and by means of the Mann-Whitney U test in case of non-normal distribution. *P*-values of ≤0.05 will be considered statistically significant.

Categorical data will be displayed as numbers and percentages and analysed using a Chi-square test.

Investigational site, surgical procedure, age, and score on the eHealth literacy test will be taken into account as covariates in the adjusted analyses. Intended subgroup analyses will be conducted for the surgical procedure.

To estimate the primary outcome, the overall average compliance to the selected active ERAS elements, the elements will be scored as ‘completed’ or ‘not completed’ (dichotomous), as described by the ERAS protocol. For example, the urine catheter should be removed on day one after surgery. If this could not take place, the element was scored as ‘not completed’. For both the intervention and control groups, the proportion of completion of each individual active element will be calculated. The overall compliance is an average of the individual completion percentages. The protocol compliance is a continuous variable and will be reported as mean and standard deviation in case of normal distribution and as median and 95% confidence intervals in case of non-normal distribution.

The relation of the use of the application with the secondary outcomes mentioned in Table 5 will be examined with a linear regression.

### Prognostic factors

Preoperative baseline characteristics will be collected (sex, age, ASA classification, Body Mass Index (BMI), smoking, alcohol intake, Karnofsky scores, co-morbidity, indication for surgery and eHealth literacy will be assessed standardized).

### Potential confounders

Major per- and postoperative events, such as complications during surgery or a prolonged hospital stay due to complications in the postoperative course, readmission, or re-intervention within the 30 days follow up period after surgery, are considered as potential confounders. Direct caregivers, such as the nurse, are instructed to register potential confounders in the EHR. The coordination researcher will screen each participant for the EHR for per- and post-operative complications after the follow up period is completed.

### Trial discontinuation and withdrawal

When a trial participant experiences unmanageable negative feelings caused by the use of the APPtimize application, such as anxiety or physical discomfort, discontinuation of participation to the APPtimize trial will be recommended. Patients are informed of their right to withdraw from the trial without explanation at any time. Withdrawn participants will be asked if data about their hospital admission can be collected and they will be asked to sign an informed consent if they agree to this data collection. Data collection of withdrawn participants consists of: general patient characteristics, eHealth literacy assessment, disease related characteristics and postoperative data.

### Dissemination of trial results

The results of the APPtimize trial will be disseminated by publication in peer-reviewed scientific journals and by presentations at scientific conferences. Also, patient organizations with an interest in benign or malignant colorectal disease will be informed about the results of the trial.

## Discussion

Nowadays, it is strongly recommended that patients should be enabled to self-manage their health and also be able to participate actively in their care pathway. It is acknowledged that improving the ERAS protocol without actively involving patients into their care pathway is difficult [11]. Although the perioperative care for patients undergoing colorectal surgery improved after implementing the ERAS protocol, patient involvement can still be improved. To engage patients and maximize the potential of the ERAS protocol, innovative eHealth solutions have great potential [19, 20, 28]. To our knowledge, the APPtimize trial is the first RCT that combines an activity tracker with an interactive mobile application that truly focuses on patient education, participation and activation in order to enhance postoperative recovery. This is also the first study investigating the effect of an eHealth intervention to improve and assess the ERAS protocol in patients undergoing major abdominal surgery.

Cook et al. conducted the first cohort study using an activity tracker to monitor patients after cardiac surgery [21]. The study showed that the postoperative steps significantly influenced the LOS. In the recently published study of Van der Meij et al., the effect of an online personalized eHealth-care program on return to normal activities after surgery was evaluated [29]. The Patient-Reported Outcomes Measurement Information System Physical Function (PROMIS-PF) item bank was used to assess return to normal activities after surgery. The results showed that the personalized eHealth-care program had a significant effect on time until return to normal activities after surgery - 21 days (IQR 17–25) for participants in the intervention group versus 26 days (20–32) for participants in the control group. In our opinion, the APPtimize trial combines the best of the previously-mentioned studies and adds a tailored approach to the individual care pathway by setting a personalized postoperative daily step goal based on mean preoperative daily step count estimated in the 7 days prior to surgery. We do acknowledge that monitoring daily activity until 21 days after surgery is outside the scope of the ERAS protocol. However, assessing the effects of the use of eHealth mobile on daily activity on longer term is a unique opportunity. By adding ‘patient-reported outcome measures’ the patient’s subjective perception of the effect of the application on the postoperative outcomes is assessed as well.

Potential bias could occur due to the diversity of the study participants regarding to their age, diverse socio-economic status, and different types of colorectal disease. For example, one might suggest that elderly participants could affect study results as some will not be able to work with the application and activity tracker correctly. In our belief, elderly participants should be very capable of using the mobile application and activity tracker. However, to minimize these effects of a selection bias, we chose a randomized study design. Finally, it is reckoned that by distributing activity trackers to patients of the control group and letting them fill out a daily diary, a more active participation to the ERAS care pathway could occur. Subsequently, this will result in a decreased compliance difference between the two study groups. However, if a significant difference of the primary outcome compliance will be found, it even further emphasizes the clinical relevance of a patient-centred mobile application.

## Conclusion

We aimed to demonstrate that the proposed APPtimize mobile application has the potential to increase involvement of patients into the ERAS care pathway and, therefore, encourages patients to be more in control of their own health. By actively involving patients into the ERAS care pathway, positive effects are expected of the compliance to the active ERAS protocol elements. Through enhancement of the active ERAS elements, postoperative outcomes, such as LOS and complication rates, might benefit as well.

## Additional file

Additional file 1: “Functional design of the APPtimize application”. NB Written permission to publish content relating to the APPtimize mobile application was obtained from the designer. (PDF 1491 kb)

## Data Availability

Data sharing is not applicable to this article as no datasets were generated or analysed during the current study. The future APPtimize trial data will be available from the corresponding author on reasonable request.

## Abbreviations

CRF: Case Report Forms
ERAS: Enhanced Recovery After Surgery
EHR: Electronic Health Record
LOS: Length Of Stay in hospital
NRS: Numeric Rating Scale
PROMs: Patient Reported Outcome Measures
RCT: Randomized Controlled Trial
UMC: University Medical Centre
