# Supplementary material for: Improving enhanced recovery after surgery (ERAS): ERAS APPtimize study protocol, a randomized controlled trial investigating the effect of a patient-centred mobile application on patient participation in colorectal surgery
Source: BMC Surg. 2019 Sep 2;19:125. doi: 10.1186/s12893-019-0588-3 (PMC6719362; doi:10.1186/s12893-019-0588-3)
Supplement: Supplementary file 1 — “Functional design of the APPtimize application”. NB Written permission to publish content relating to the APPtimize mobile application was obtained from the designer. (PDF 1491 kb) [file 12893_2019_588_MOESM1_ESM.pdf]

everywhereIM
Paasheuvelweg 25
Wing 5D
1105 BP Amsterdam

Functioneel ontwerp

AMC
ERAS-APPtimalisatie

03600-19

1.0 Inleiding

Dit document bevat het functioneel ontwerp van de ERAS-APPtimalisatie-app van het AMC.

Het exacte design zal in de volgende fase, de design fase, worden behandeld. Nu staan de functionaliteiten centraal. Op iedere pagina is er ruimte voor eventuele opmerkingen en een paraaf. Op de laatste pagina vragen we u een handtekening te plaatsen. Na ondertekening van het functioneel ontwerp wordt er overgegaan op de design- en softwareontwikkelingsfase.

We zijn uiteraard bereikbaar voor vragen en/of opmerkingen.

Marilou Jansen, MD

Projectmanager
everywhereIM

Mail: marilou@everywhereIM.com
Web: www.everywhereIM.com
Adres: everywhereIM
Paasheuvelweg 25
Wing 5D
1105 BP Amsterdam

2.0 Inhoudsopgave

1.0 Inleiding 2
3.0 Schetsen 4
3.1 Splashscreen 4
3.2 Eerste keer inloggen 5
3.4 Reguliere inlog 7
3.5 Informed consent 8
3.6 Registratieformulier 9
3.7 Hoofdscherm 10
3.8 Tijdlijn 18
3.9 Informatie 27
3.10 Dashboard 29
3.11 Instellingen 30
4.0 Handtekening voor akkoord 42
5.0 Disclaimer 43
Bijlage 1 44
Bijlage 2 45

3.0 Schetsen

3.1 Splashscreen

Omschrijving:
Dit scherm wordt kortdurend getoond terwijl de app aan het laden is. De gebruiker hoeft niets te doen om naar het volgende scherm te gaan.

Onderdelen:
1. Dit scherm kan volledig naar wens worden ingericht. Hier wordt de titel van de app getoond.
2. Logo's kunnen worden getoond. Dit zal worden besproken met AMC-voorlichting/het online team.
3. Logo VUmc, zij zullen vanaf de start van de trial participeren. Ook in het VUmc zal contact worden gelegd met voorlichting.

Opmerkingen:

____

____

____

Paraaf: __________

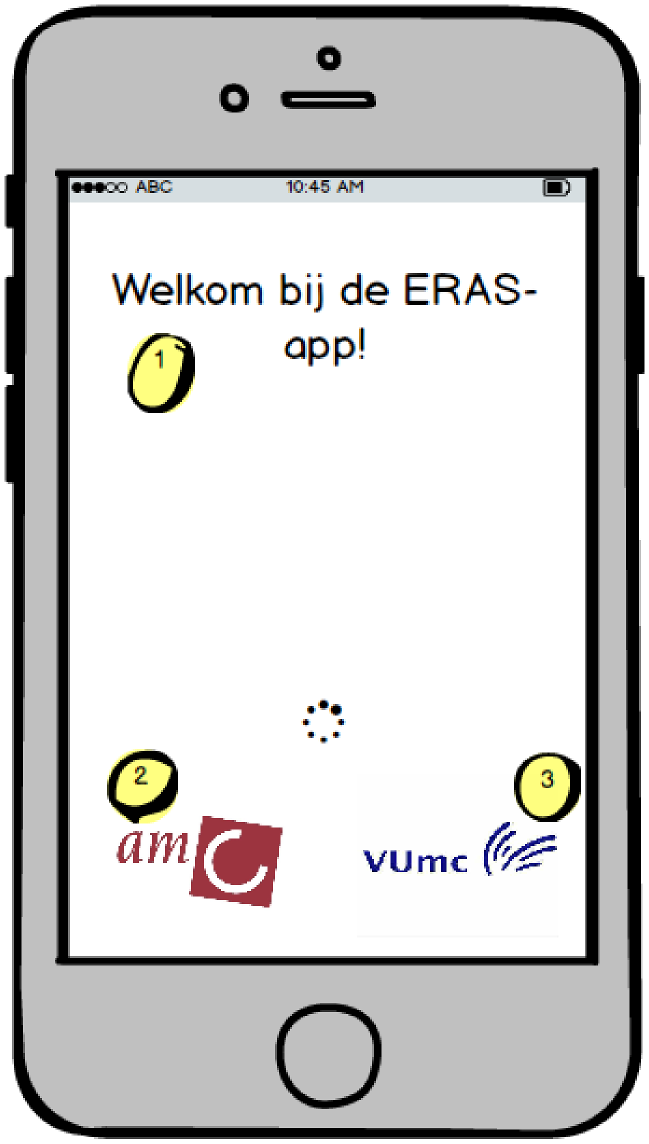

3.2 Eerste keer inloggen

Omschrijving:
Als gebruikers voor de eerste keer na het downloaden de app openen wordt dit scherm getoond. Dit is eenmalig.

Onderdelen:
1. Studiepatiënten worden door de onderzoekers aangemeld. Vanwege de noodzaak om veilig data te moeten kunnen versturen is "cross-referencing" belangrijk. De inlog voor studiepatiënten verloopt daarom via een aparte route, zie hiervoor 3.3.
2. Deze optie kan worden ingebouwd, maar is niet noodzakelijk. Als het de wens is om de app vrij in de store beschikbaar te stellen is het belangrijk dat ook andere gebruikers toegang hebben, zonder dat hun data opgeslagen wordt of op wat voor wijze dan ook verward kan worden met data van de studie-patiënten.

Opmerkingen:

____

____

____

Paraaf: ___________

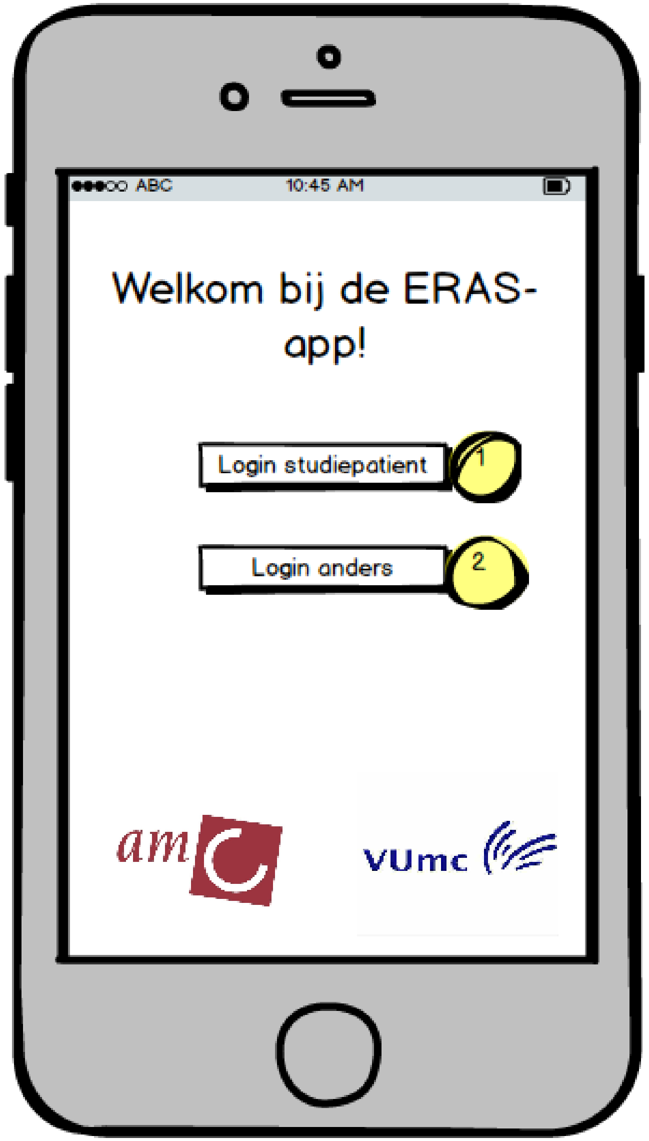

3.3 Registratie studiepatiënt

Omschrijving:
Nadat de onderzoeker de proefpersoon heeft toegevoegd in het dashboard op basis van naam en emailadres wordt een unieke code gegenereerd. Deze code is beperkt geldig. Het emailadres wordt direct *ge-encrypt,* maar moet wel worden opgeslagen voor als de gebruiker een nieuw wachtwoord moet opvragen. De gegenereerde code wordt per email en/of mondeling aan de proefpersoon verstrekt.

Onderdelen:

1. Dit is een vrij invoerveld, waar de code kan worden ingevoerd.
2. Met deze knop wordt de invoer bevestigd.

Opmerkingen:

____

____

____

Paraaf: _______________

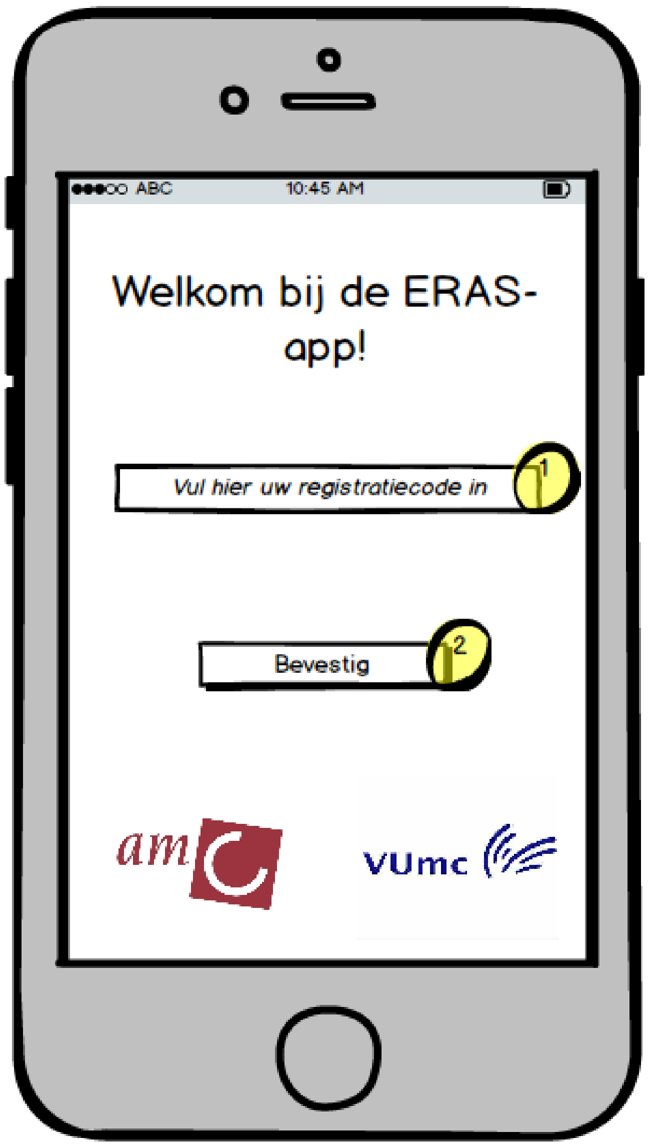

3.4 Reguliere inlog

Omschrijving:
Nadat de proefpersoon zich voor de eerste keer heeft geregistreerd, moet voortaan via dit scherm worden ingelogd. Elke keer als de app wordt uitgeschakeld (ook niet meer op de achtergrond actief), moet opnieuw worden ingelogd. Dit is noodzakelijk omdat een aantal persoonsgegevens op het device blijven opgeslagen, zoals het type operatie en de operatiedatum.

Onderdelen:
1. De gebruiker kan inloggen met het zelfgekozen wachtwoord
2. De gebruiker kan een pincode aanmaken, dit zal verder worden toegelicht bij "Instellingen". Hiermee kan versneld worden ingelogd.
3. Als de gebruiker zijn of haar wachtwoord is vergeten, kan via deze link een nieuw wachtwoord worden aangevraagd.
4. Zodra wachtwoord of pincode is ingevoerd, kan met deze knop worden ingelogd.

Opmerkingen:

____

____

____

Paraaf: ______________

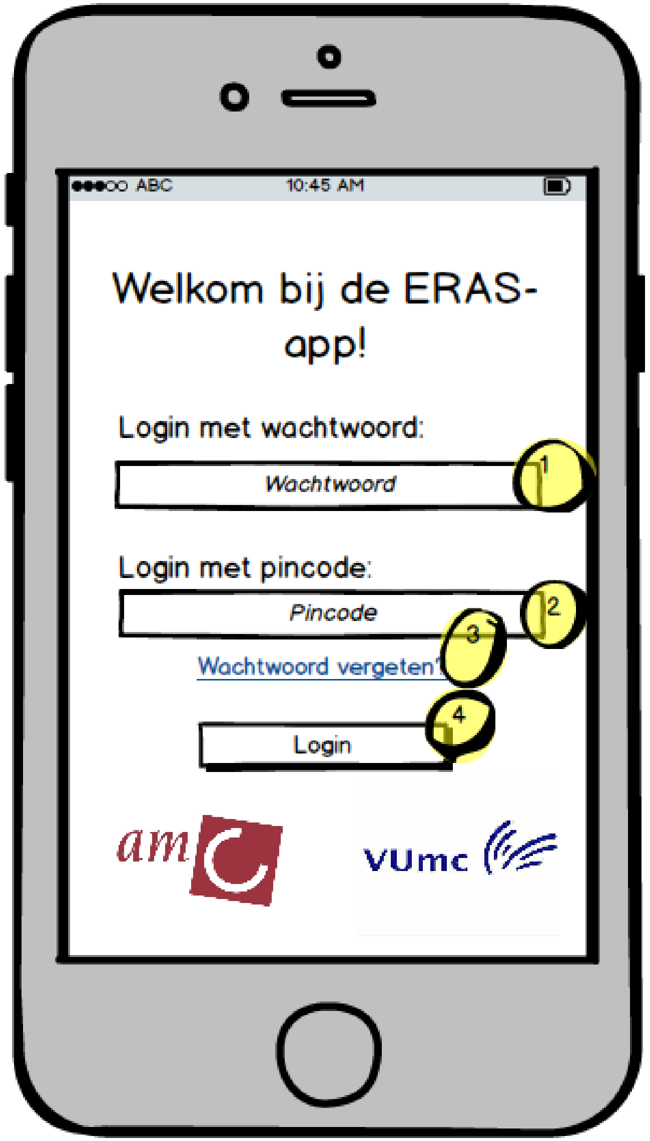

3.5 Informed consent

Omschrijving:
De eerste keer dat de een studiepatiënt de app opent wordt dit scherm getoond. Doormiddel van ondubbelzinnige "opt-in" wordt hier om het informed consent van gebruiker gevraagd. Deze informatie is altijd via de instellingen van de app op te roepen en te wijzigen.

Onderdelen:

1. Dit is een check-box, dat betekent dat meerdere antwoorden mogelijk zijn. Alleen als alle vier de *boxes* zijn aangevinkt wordt dit gezien als een geldig informed consent. Bij het menu "Instellingen", dat is uitgewerkt in paragraaf 3.11, kan het informed consent of een deel daarvan weer worden ingetrokken.
2. Met deze knop worden bovenstaande keuzes bevestigd. Het proces voor een Privacy Impact Assessment (PIA) is reeds in gang gezet.

Opmerkingen:

____

____

____

Paraaf: ______________

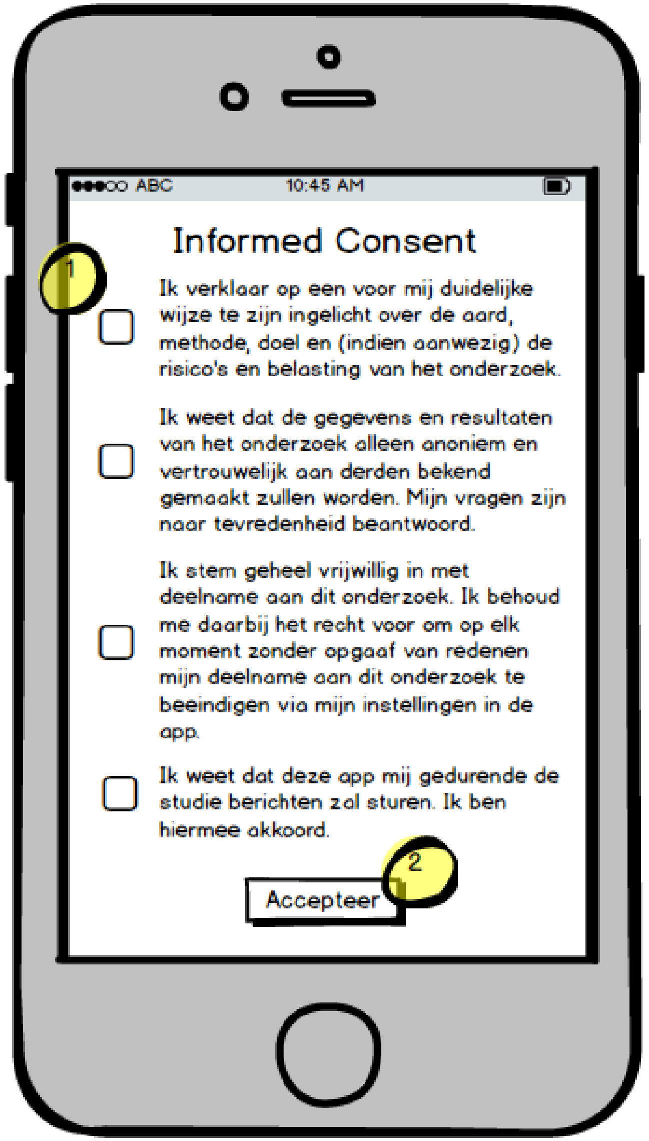

3.6 Registratieformulier

Omschrijving:
Een belangrijk onderdeel van de app is de tijdlijn. Deze wordt gegenereerd op basis van de operatiedatum die op deze pagina ingevuld kan worden. Tevens kunnen een aantal basisvoorkeuren worden ingesteld. Ook dit menu is altijd vanuit de app te bereiken en antwoorden kunnen desgewenst gewijzigd worden.

Onderdelen:
1. Het type operatie kan worden aangegeven. Dit wordt geregistreerd omdat het een confounding variabele kan zijn. Zie bijlage 1 voor de gekozen terminologie.
2. De datum van de operatie is cruciaal voor het genereren van de tijdlijn. Zie voor de uitwerking hiervan 3.11.1.2.
3. Hier kan een gebruikersnaam worden gekozen, ter personificatie van de app.
4. Hier kan de gebruiker een wachtwoord naar keuze registeren. Voor wachtwoord instellen en wijzigen zie 3.11.3.1.
5. Hier wordt het wachtwoord bevestigd.
6. Hier geeft de gebruiker toestemming voor het feit dat Google Analytics op de achtergrond gegevens verzamelt over het gebruik van de app.
7. Hier kan een infobutton over Google Analytics worden opgeroepen, zie 3.11.3.4.
8. Met deze knop wordt de invoer bevestigd.

Opmerkingen:

___

___

___

Paraaf: ______________

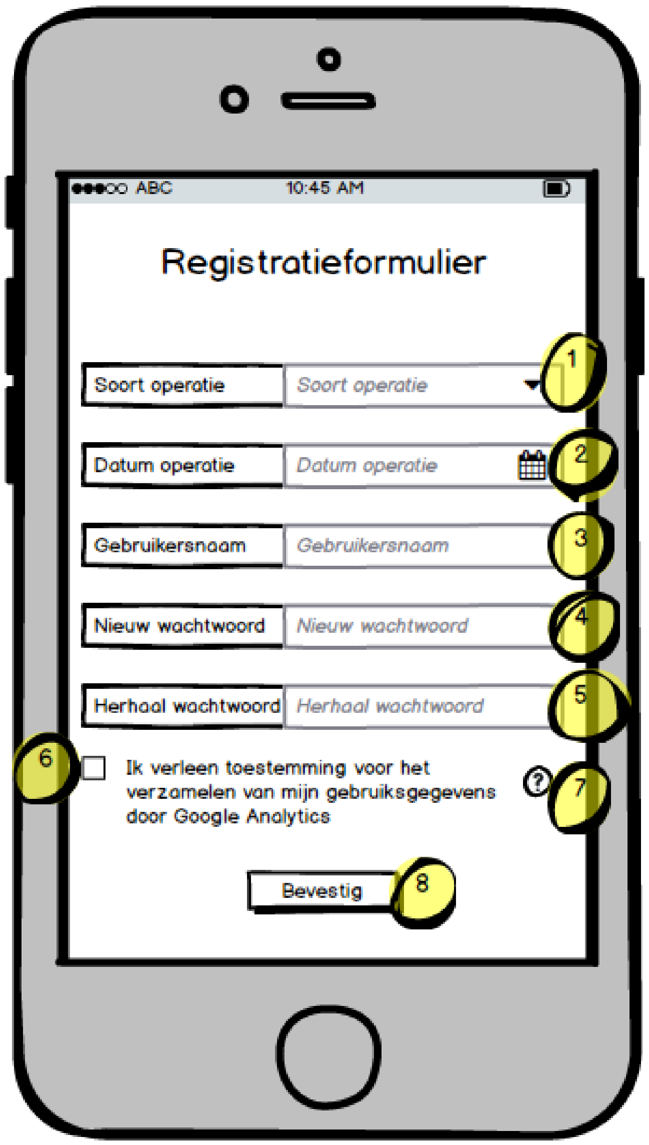

3.7 Hoofdscherm

Omschrijving:
Als alle bovenstaande gegevens zijn ingevoerd (dit is eenmalig), wordt op basis van deze gegevens de persoonlijke tijdlijn gegenereerd. Na invoeren wordt het volgende scherm getoond. Dat wat wordt getoond is op basis van de afwijking van de tijdlijn (- of + zoveel dagen).
Als de gebruiker de app meer dan 3 weken voor de operatie al heeft gedownload, start deze in fase 1. De nummering is uiteraard aan te passen.
Fases die nog niet van toepassing zijn, worden zichtbaar in de app zodra zij dat op basis van de tijd wel worden.

Onderdelen:
1. Hier is het basisoverzicht van de tijdlijn zichtbaar.
2. De bottom toolbar bevat 4 iconen. Dit icoon (gemarkeerd met 2) leidt naar de home pagina. Omdat de gebruiker zich daar nu bevindt, is dit icoon disabled, wat blijkt uit de afwijkende kleur.
3. Deze knop leidt naar een overzicht van de informatie, zie paragraaf 3.9
4. Deze knop leidt naar het dashboard, zie paragraaf 3.10
5. Deze knop leidt naar de instellingen, zie paragraaf 3.11
6. Het hamburgermenu linksboven is vanuit de submenu's altijd zichtbaar, vanaf hier kan de gebruiker snel naar andere onderdelen van de app navigeren. De onderdelen van het menu worden toegelicht in paragraaf 3.7.1

Opmerkingen:

____

____

____

Paraaf: ______________

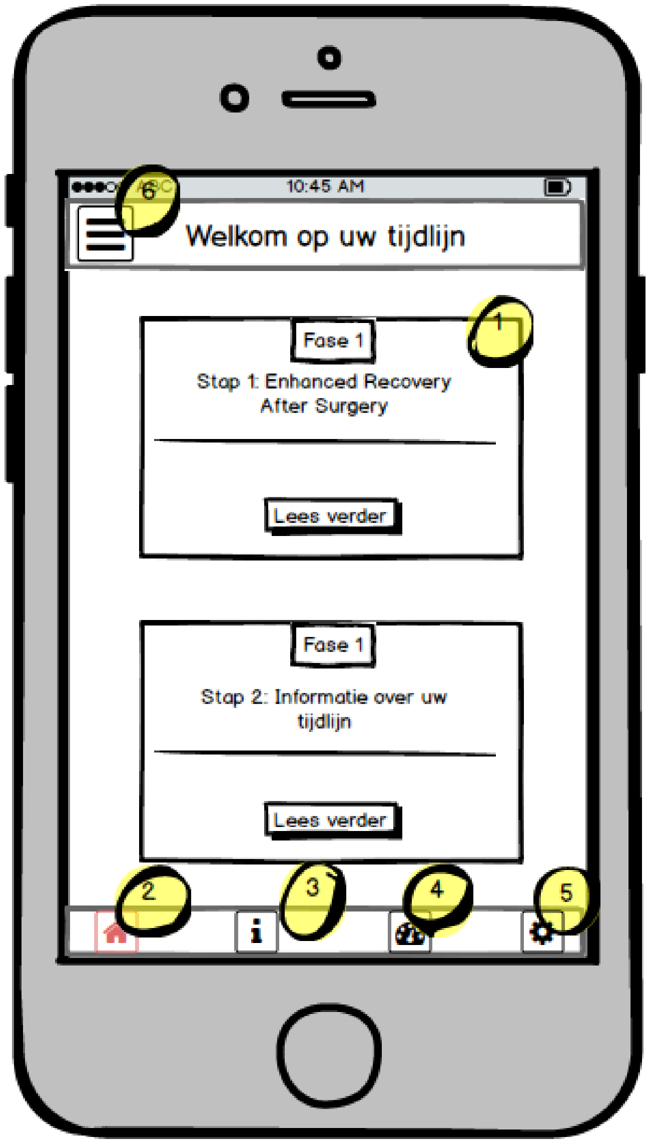

3.7.1 Hamburgermenu

Omschrijving:
In deze afbeelding worden de elementen van het hamburgermenu weergegeven.

Onderdelen:
1. De informatie pagina wordt verder uitgewerkt in paragraaf 3.9
   De pijltjes staan hier naar rechts, dat betekent dat als je erop klikt, opent zich een submenu. Zie paragraaf 3.7.1.1
2. Dit submenu is uitgewerkt in paragraaf 3.7.1.2
3. Deze pagina is uitgewerkt in 3.7.1.3

Opmerkingen:

____

____

____

Paraaf: ____________

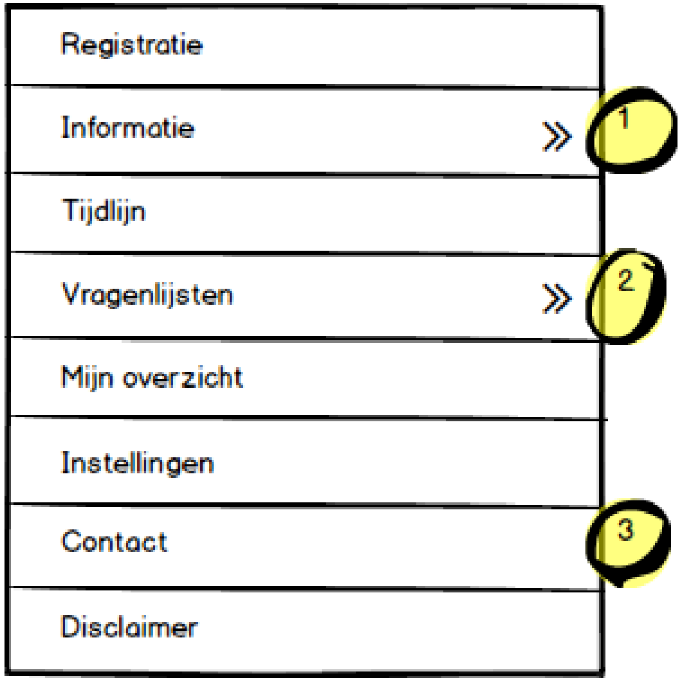

3.7.1.1 Hamburgermenu - Informatie

Omschrijving:
In deze afbeelding wordt het submenu "Informatie" getoond. De informatie is geordend per fase.

Onderdelen:
1. Als een submenu wordt uitgeklapt is dat zichtbaar aan de pijlen bij het onderdeel. Door nogmaals te klikken wordt het menu weer ingeklapt.

Opmerkingen:

___

___

___

Paraaf: ______________

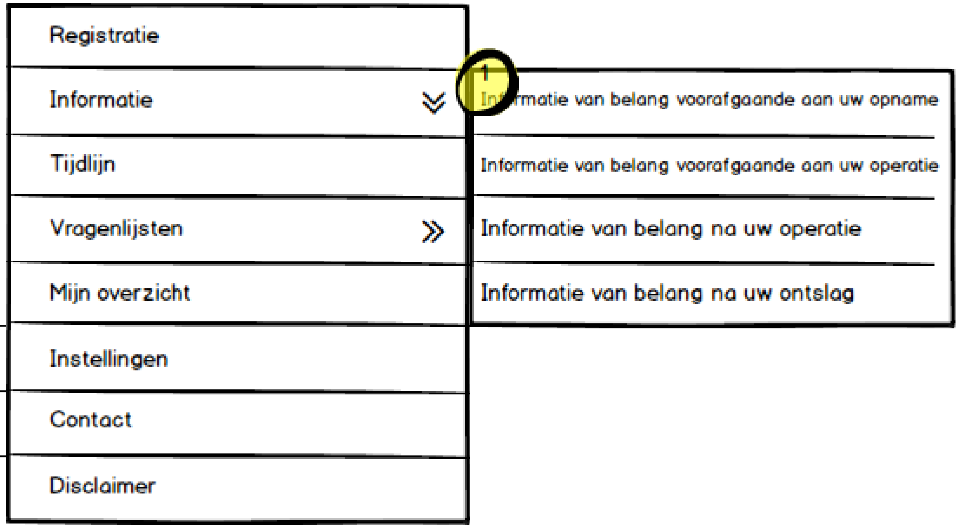

3.7.1.2 Hamburgermenu – Vragenlijsten

Omschrijving:
In deze afbeelding wordt het submenu "Vragenlijsten" getoond. Ook de vragenlijsten ten behoeve van dataverzameling kunnen digitaal worden ingevoerd.

Onderdelen:
1. Als een submenu wordt uitgeklapt is dat zichtbaar aan de pijlen bij het onderdeel. Door nogmaals te klikken wordt het menu weer ingeklapt.
2. Hier staat kort informatie over waarom in deze app vragenlijsten beschikbaar komen
3. Hier staan voorbeelden van vragenlijsten die aan de patiënt worden voorgelegd. Deze komen beschikbaar wanneer relevant, op basis van de operatiedatum.

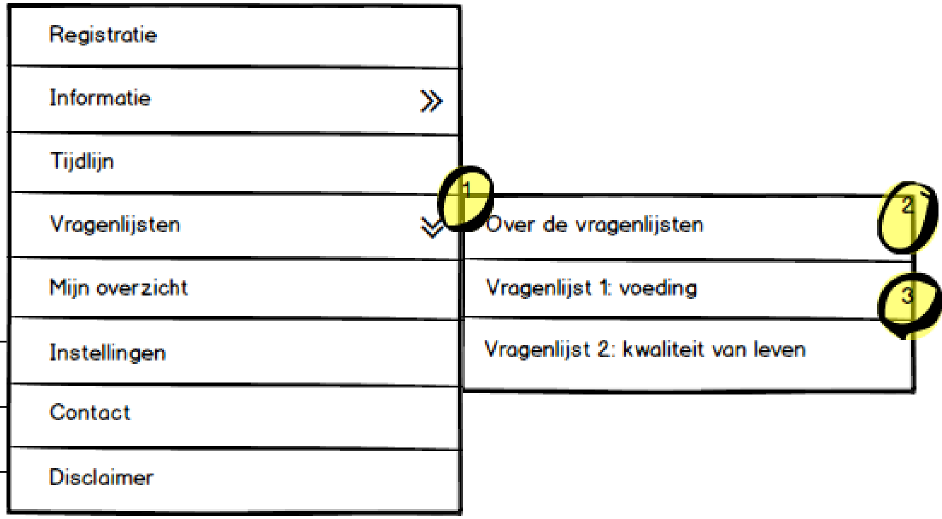

Opmerkingen:

Paraaf: ______________

3.7.1.3 Contact

Omschrijving:
De gebruiker kan onder het kopje "Contact" alle benodigde contactgegevens vinden.

Onderdelen:
1. Alle kopjes met een telefoon-icoontje verwijzen naar een pagina met contactgegevens. Zie 3.7.1.3 Contact – Telefoon.
2. Alle kopjes met een website-icoontje hebben de functie om doorverwezen te worden naar een website waar de informatie te vinden is.
3. Ook zijn er routewijzers die de gebruiker vanaf de ingang van het betreffende ziekenhuis naar de polikliniek of verpleegafdeling chirurgie helpen.

Opmerkingen:

____

____

____

Paraaf: ____________

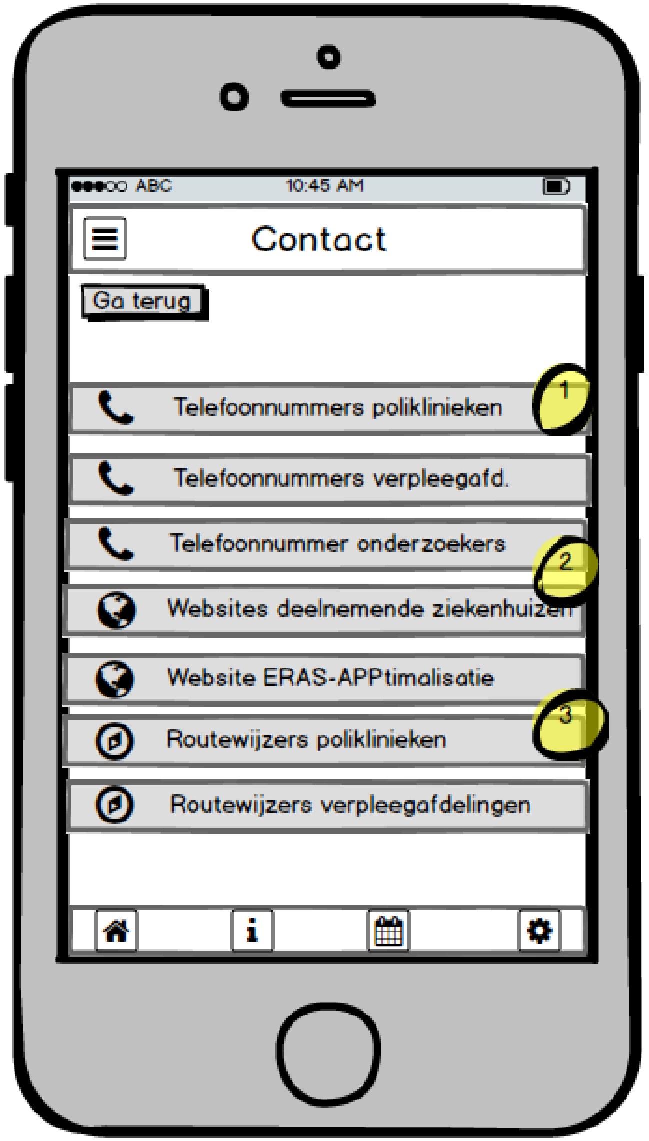

3.7.1.3 Contact-Telefoon

Omschrijving:
De gebruiker kan onder het kopje “Contact” alle benodigde contactgegevens vinden.

Onderdelen:
1. Via deze knop gaat de gebruiker terug naar het scherm op de vorige pagina.
2. Hier worden kort de gegevens van het betreffende ziekenhuis en de bereikbaarheid genoemd
3. Via deze knop kan rechtstreeks gebeld worden, zie ook pagina 16.
4. Hier wordt de locatie van het ziekenhuis weergegeven.

Opmerkingen:

____

____

____

Paraaf: ______________

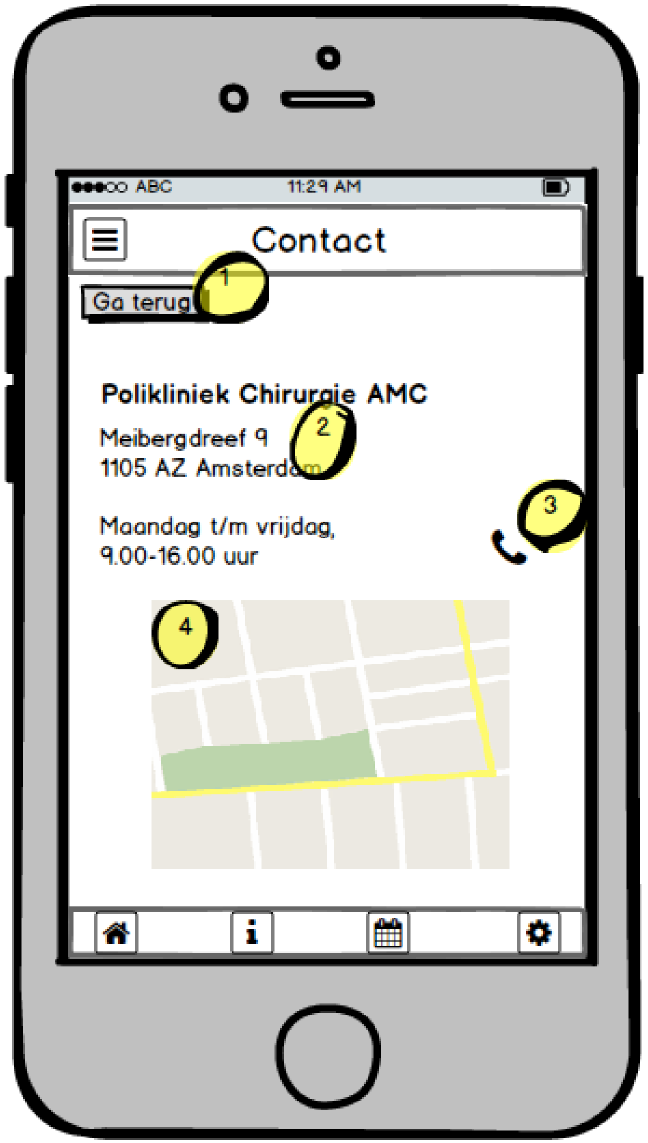

3.7.1.3. Direct bellen

Omschrijving:
Als gekozen wordt voor direct bellen wordt eerst nog een waarschuwing en het volledige nummer getoond.

Onderdelen:
1. Als de gebruiker alleen het nummer wil noteren, kan de gebruiker het bellen hier annuleren.
2. Hier kan het bellen worden bevestigd. Het gesprek wordt automatisch gestart.

Opmerkingen:

___

___

___

Paraaf: _______________

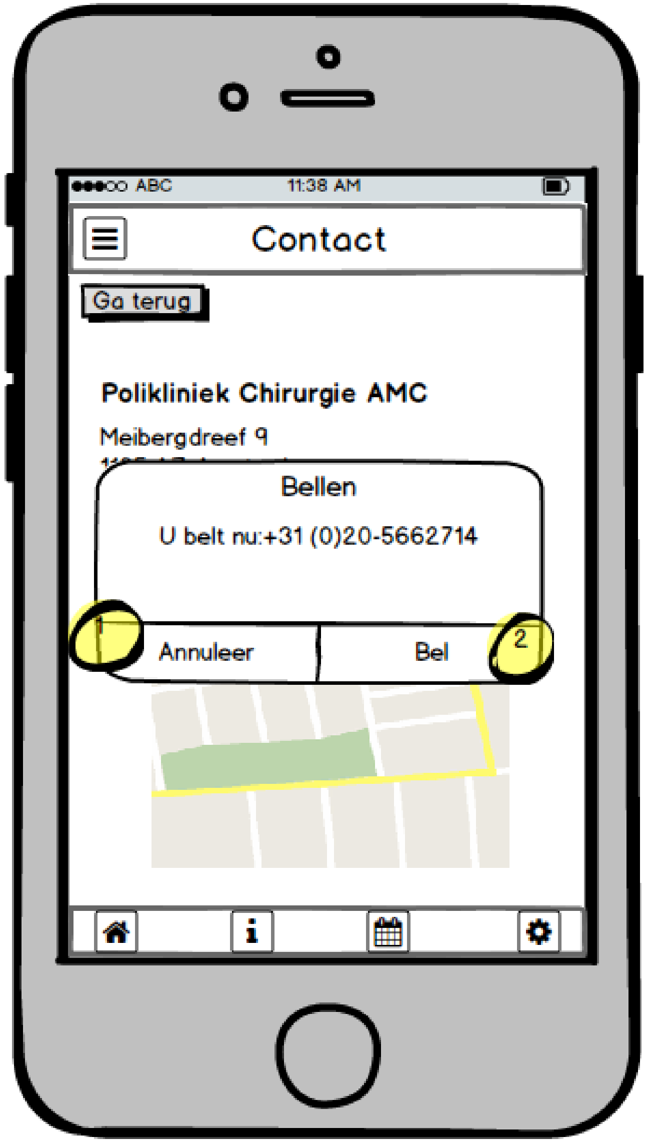

3.7.1.3 Contact-Routewijzers

Omschrijving:
De routewijzer kan in de app getoond worden om de gebruiker naar de juiste polikliniek of verpleegafbeelding te wijzen.

Onderdelen:

1. Hier kan de gebruiker de pagina weer verlaten.
2. Hier kan een afbeelding van de plattegrond worden getoond. De gebruiker kan via locatievoorzieningen op de kaart worden getoond, maar helaas niet op een gedetailleerde plattegrond van het ziekenhuis. Er wordt daarom voor gekozen om alleen een statische plattegrond te tonen.

Opmerkingen:

____

____

____

Paraaf: ______________

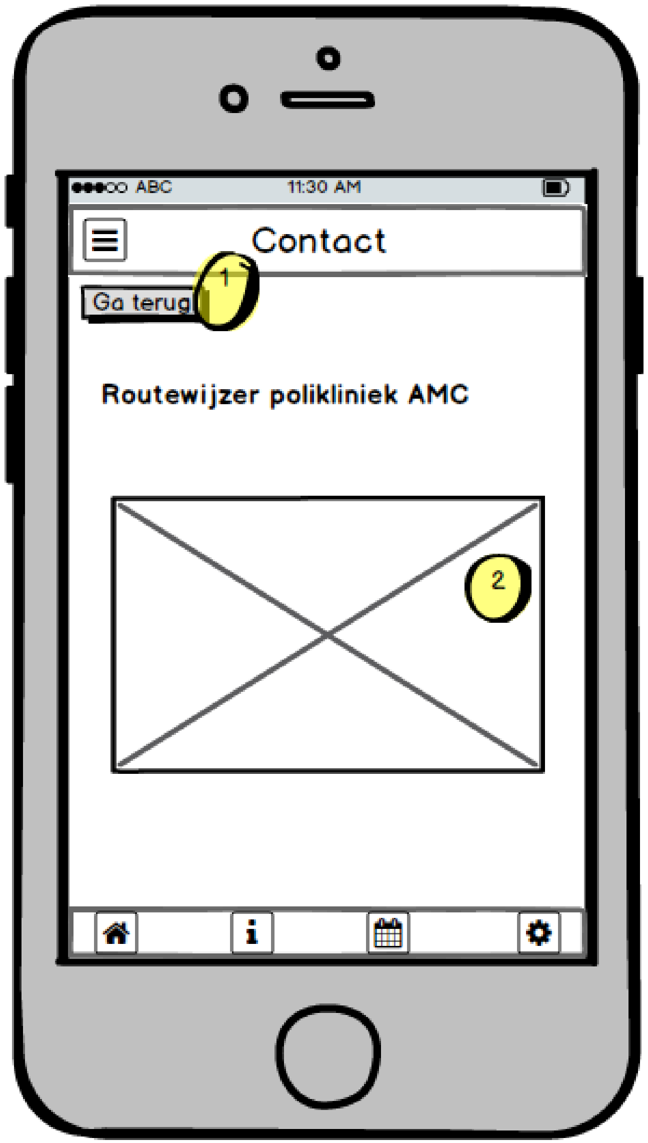

3.8 Tijdlijn

3.8.1 Meer dan 3 weken tot de operatie

Omschrijving:
Een van de doelen van de app is om de gebruiker te informeren over het ERAS zorgpad, met informatie die getimed wordt aangeboden. De elementen op de tijdlijn die hier worden weergegeven zijn informatief, met een afwijkende vorm (in dit geval een rechthoek) wordt aangegeven dat het informatieve blokken betreft.

Onderdelen:
1. Afhankelijk van de ingevulde operatiedatum wordt de fase waarin de gebruiker zich bevindt getoond. De fase zal altijd worden weergegeven, de naamgeving kan ook anders.
2. Hier staat kort het onderwerp dat in deze fase benadrukt wordt. Een rechthoekig element staat voor informeren.
3. Via deze knop kan de gebruiker verder lezen, een pagina met meer informatie wordt geopend.

Opmerkingen:

____

____

____

Paraaf: ______________

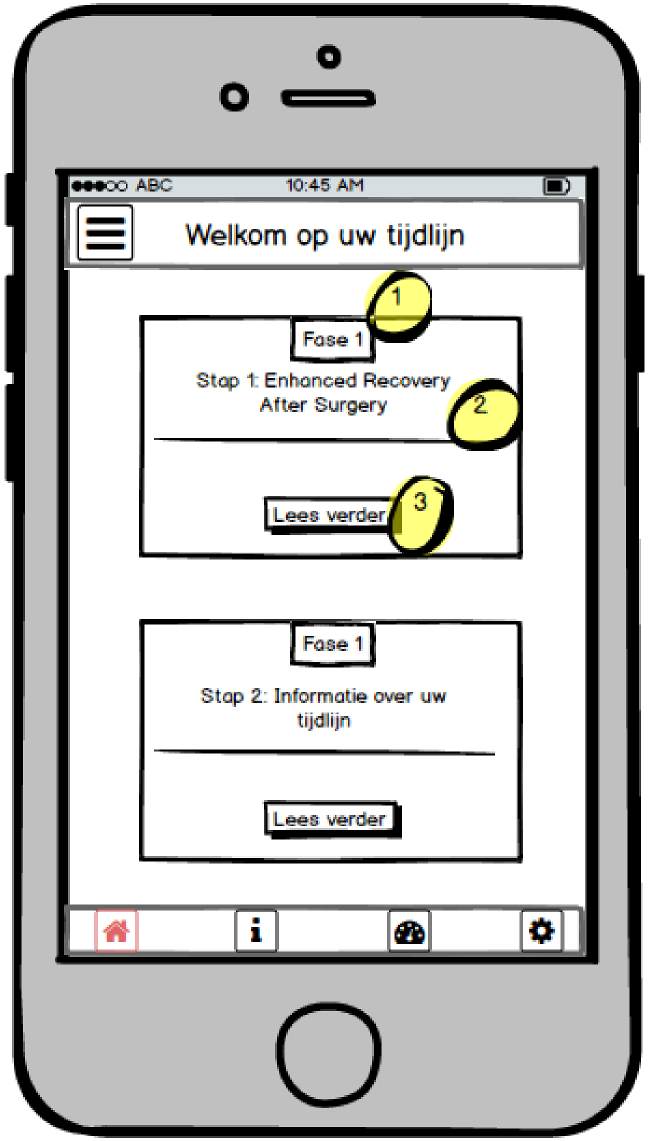

3.8.2 Tijdlijn – 3 weken tot de operatie

Omschrijving:
De gebruiker kan direct na inclusie de app downloaden, de informatie is dan al te raadplegen.
Drie weken voor de operatie verschijnt de eerste notificatie (ook als de app op dat moment niet op de achtergrond actief is) om de gebruiker op specifieke relevante informatie of vragenlijsten te wijzen. Let op: actiepunten en vragenlijsten zijn nog niet zichtbaar. Deze komen pas beschikbaar wanneer zij op basis van de tijd relevant worden.

Onderdelen:
1. In het scherm van de telefoon van de gebruiker verschijnt deze notificatie.
2. De gebruiker heeft de keuze om naar de app te gaan of om de notificatie te negeren. We gaan er nu vanuit dat de gebruiker ervoor kiest om de app te openen. In dat geval wordt de app geopend bij paragraaf 3.8.3

Opmerkingen:

____

____

____

Paraaf: ____________

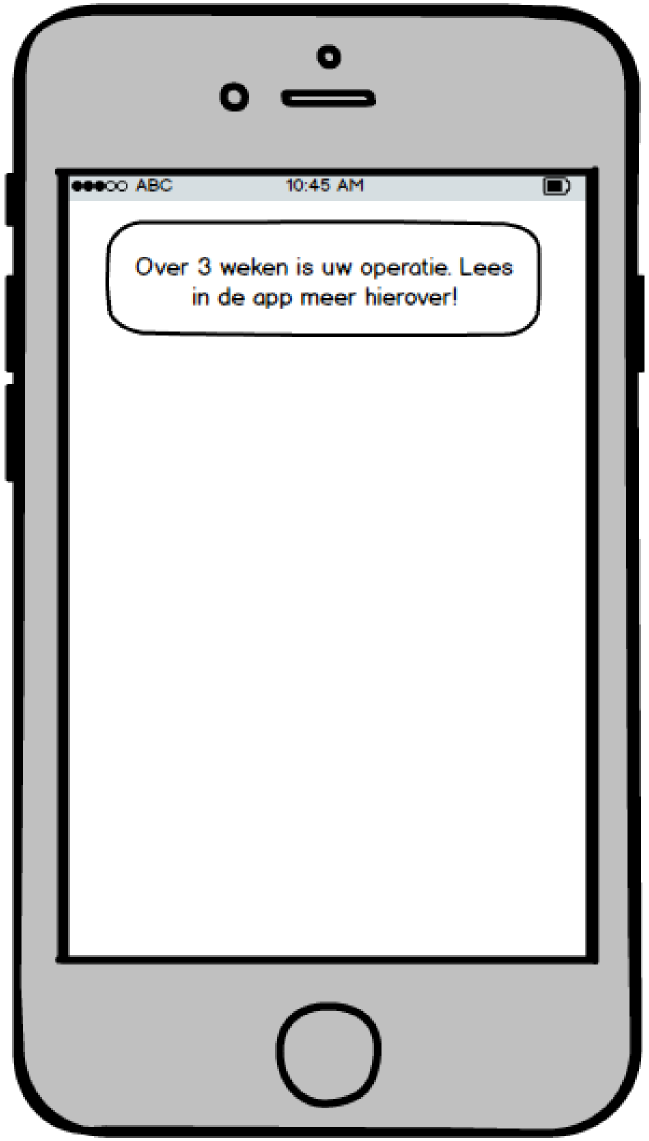

3.8.3 Drie weken tot de operatie – stap 1: vragenlijst

Omschrijving:

Als de gebruiker op “Ga naar de app” heeft gedrukt, wordt de app gestart en komt hij/zij in dit scherm terecht. Drie weken tot de operatie wordt in de app fase 2 genoemd. Als een gebruiker in een onderdeel staat, wordt dit door middel van kleur aangegeven. Hier wordt met een afwijkende vorm aangegeven dat het een element is dat actie vereist. In dit geval een door de tijd getriggerde vragenlijst. Deze vragenlijst wordt pas zichtbaar op het moment dat dit relevant wordt op basis van de tijd.

Onderdelen:

1. De cirkel staat voor een te ondernemen actie. In de cirkel staat informatie over de vragenlijsten.
2. De patiënt kan ervoor kiezen om de vragenlijsten in te vullen. Scherm 3.8.4 wordt geopend.

Opmerkingen:

____

____

____

Paraaf: ______________

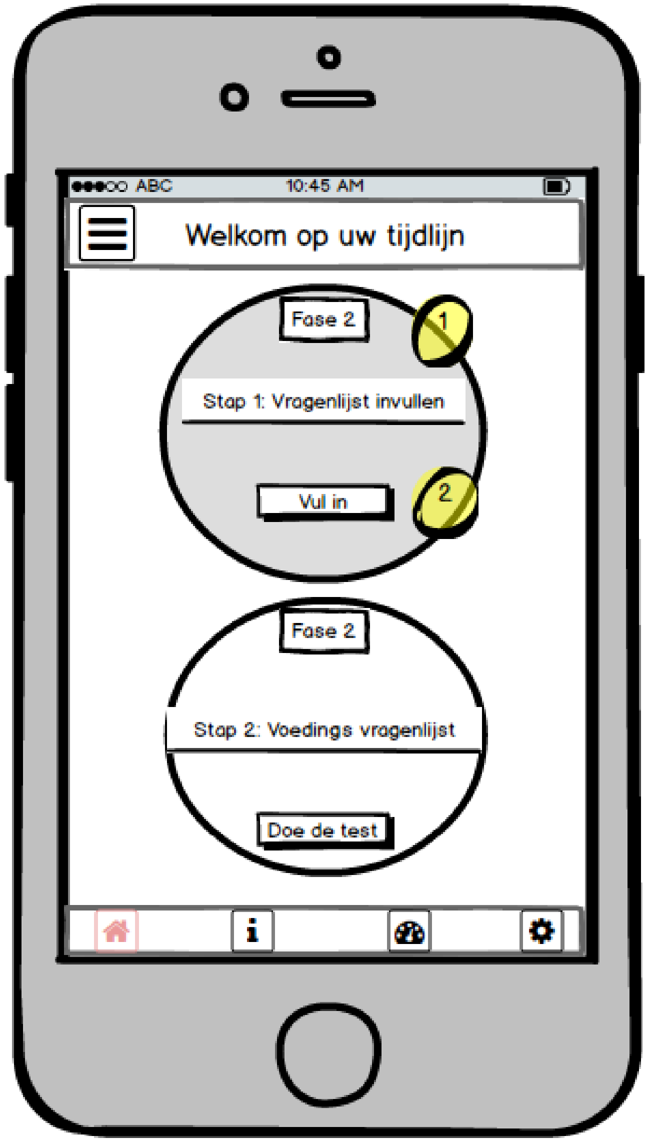

3.8.4 Drie weken tot de operatie – stap 1: uitleg onderdeel

Omschrijving:
Nadat de gebruiker op “Vul in!” heeft geklikt wordt dit scherm geopend.

Onderdelen:
1. In dit veld staat kort informatie over de vragenlijsten, waarom deze wordt afgenomen en hoeveel tijd dit zal kosten.
2. Door te scrollen/naar beneden te swipen wordt de vragenlijst geopend. In paragraaf 3.8.5 wordt een kort voorbeeld uitgewerkt.
3. Met deze knop kan de lettergrootte worden aangepast.

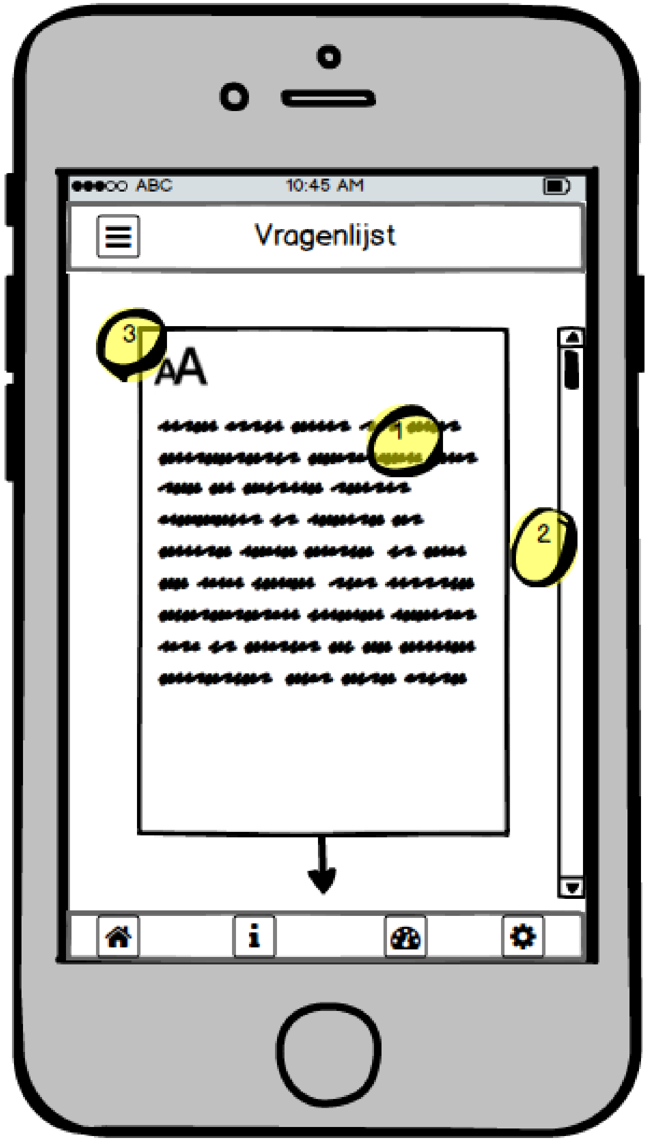

Opmerkingen:

____

____

____

Paraaf: ______________

3.8.5 Drie weken tot de operatie – stap 1: vragenlijsten invullen

Omschrijving:
Vervolgens, als de gebruiker omlaag scrolt verschijnen de eerste vragen. In deze schermen is slechts een eenvoudig voorbeeld uitgewerkt. De daadwerkelijke teksten zullen separaat worden aangeleverd.

Onderdelen:

1. De gebruiker kan het juiste antwoord aanvinken. Indien "Ja" wordt gekozen zal de gebruiker worden gevraagd dit te kwantificeren. Dit is slechts een voorbeeld.
2. Als beide vragen zijn beantwoord, kunnen de antwoorden met deze knop worden opgeslagen.

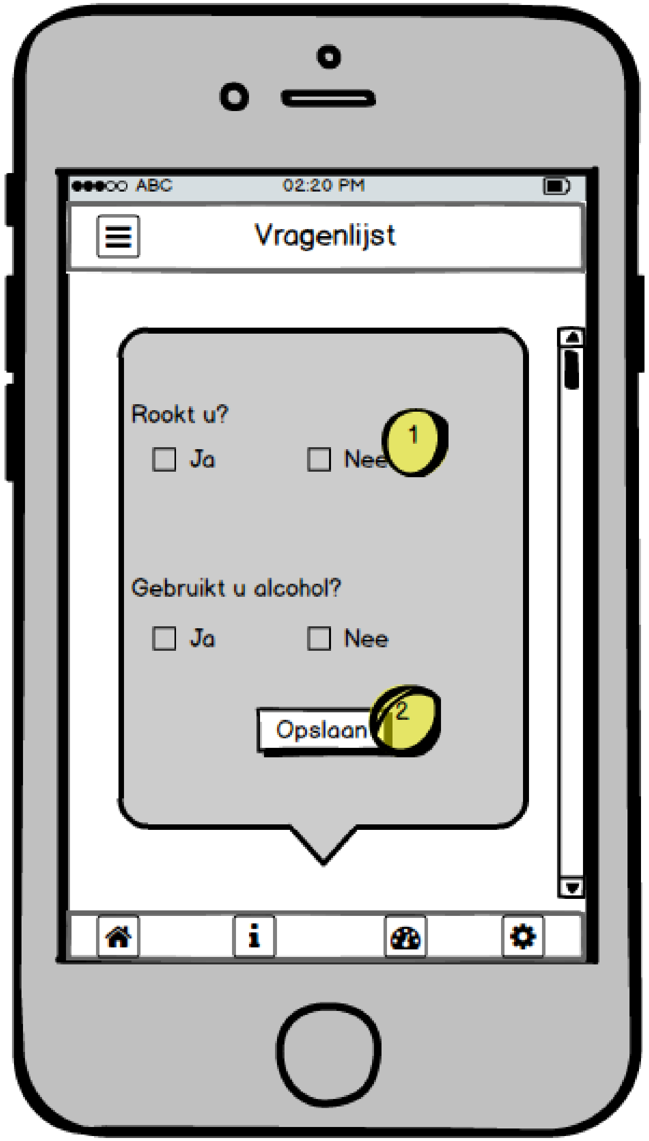

Opmerkingen:

____

____

____

Paraaf: ______________

3.8.6 Drie weken tot de operatie – stap 1: vragenlijsten invullen

Omschrijving:
In dit scherm is zichtbaar dat de antwoorden zijn ingevuld.

Onderdelen:
1. Door te dubbelklikken wordt een antwoord gedeselecteerd, door op het andere antwoord te klikken wordt dat antwoord geselecteerd.
2. Met deze knop worden de gegeven antwoorden opgeslagen.

Opmerkingen:

____

____

____

Paraaf: ______________

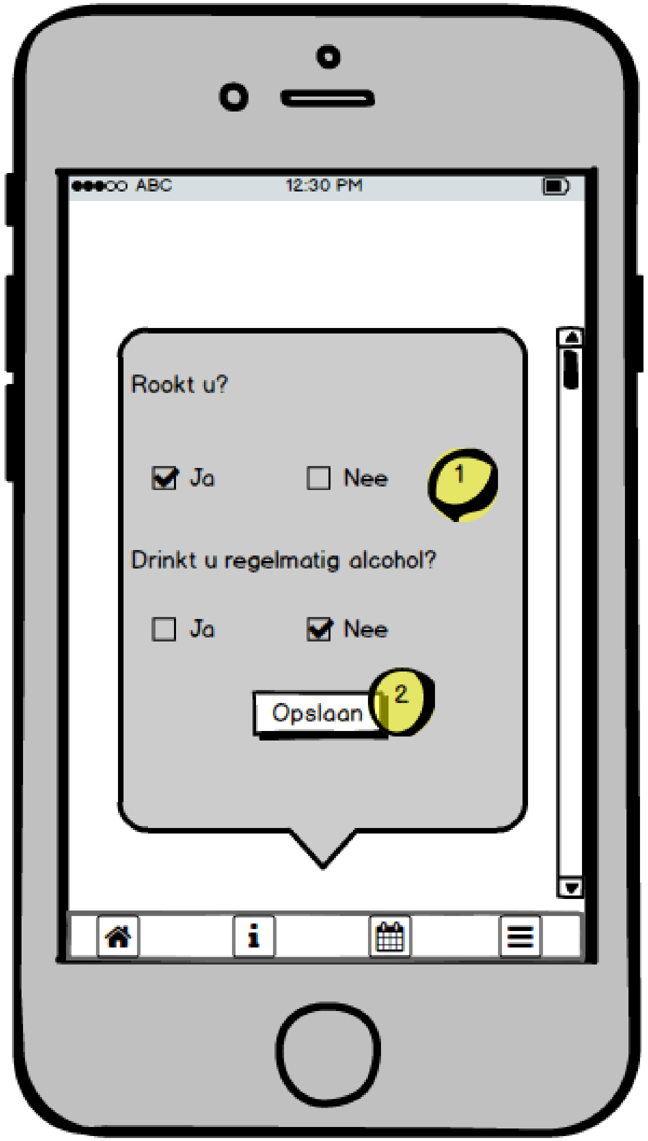

3.8.7 Drie weken tot de operatie – stap 1: vragenlijsten invullen

Omschrijving:
Voor antwoorden daadwerkelijk worden verstuurd naar de database, wordt deze *alert* getoond.

Onderdelen:
1. Hier wordt de tekst van de waarschuwing getoond.
2. Door op nee te klikken gaat de gebruiker terug naar de antwoorden en kan deze desgewenst nog wijzigen.
3. Met deze knop worden de gegeven antwoorden opgeslagen en definitief verstuurd.

Opmerkingen:

____

____

____

Paraaf: ______________

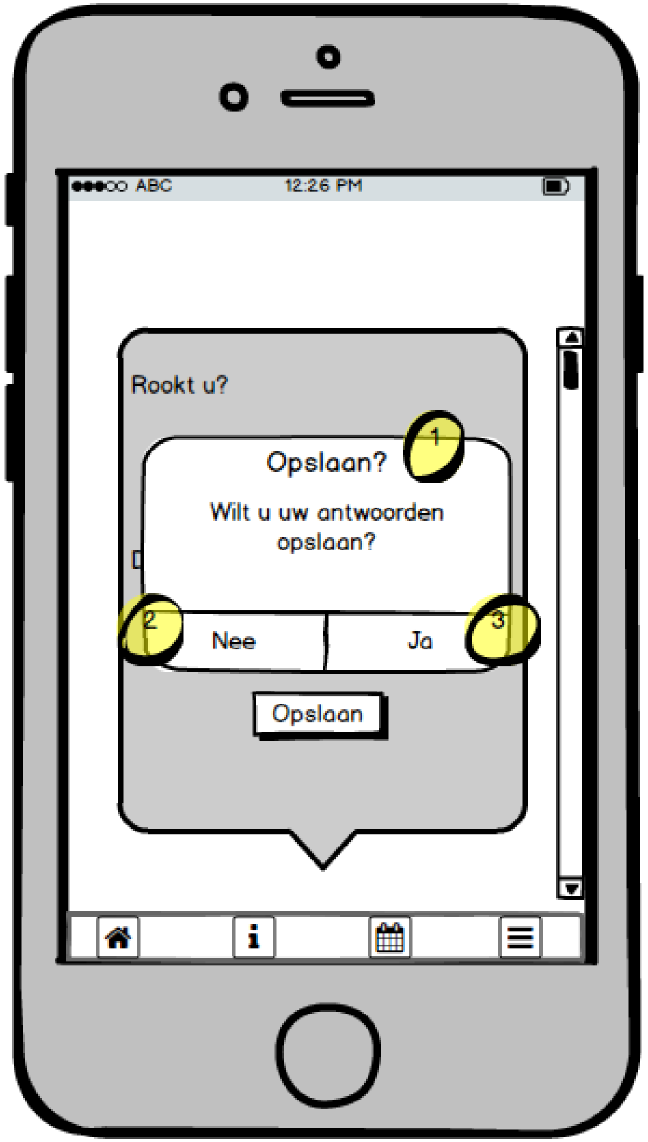

3.8.8 Drie weken tot de operatie – stap 1: vragenlijsten invullen

Omschrijving:
Indien één van de voorgaande vragen met "Ja" is beantwoord, is het soms nodig om door te vragen. Op basis van logica kan dit worden georganiseerd. In dit scherm wordt getoond hoe een antwoord kan worden gekwantificeerd. Hierbij zullen maten/eenheden zoals gehanteerd in het EPD worden overgenomen.

Onderdelen:
1. Hier kan een infobutton worden geopend voor als mensen vragen hebben over hoe dit in te vullen bij bijvoorbeeld shag of sigaren i.p.v. sigaretten of als nicotinegebruik niet dagelijks is.
2. Hier wordt een invoerveld getoond waar alleen numerieke antwoorden kunnen worden gegeven.
3. Ook hier kunnen alleen numerieke antwoorden worden gegeven.
4. Met deze knop kunnen de antwoorden worden opgeslagen. Wederom zal eerst de pop-up van 3.8.7 worden getoond.

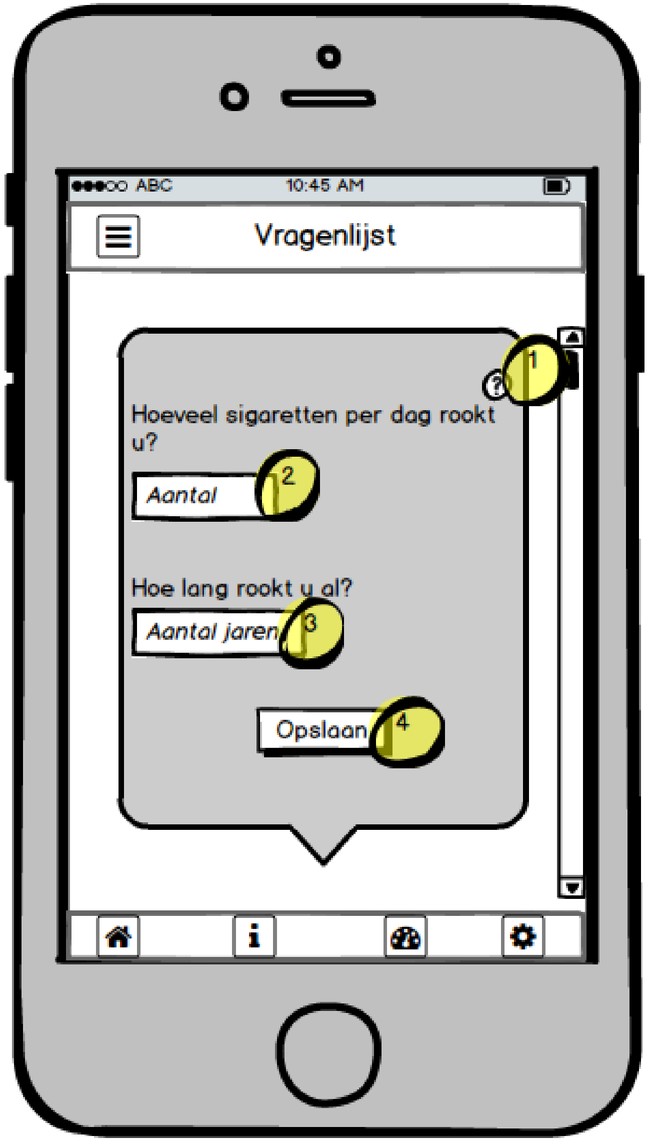

Opmerkingen:

____

____

____

Paraaf: ______________

3.8.9 Drie weken tot de operatie – stap 1: vragenlijsten invullen

Omschrijving:
De gebruiker is nu teruggekeerd naar de tijdlijn nadat de eerste stap is voltooid. Het onderdeel wordt “verzegeld” en aangegeven in een afwijkende kleur.

NB: De rest van de tijdlijn zal volgens hetzelfde concept, met ronde en rechthoekige vakken, worden uitgewerkt. De teksten zullen separaat worden aangeleverd.

Onderdelen:
1. De gebruiker kan zien dat de eerste vragenlijst is ingevuld.

Opmerkingen:

____

____

____

Paraaf: _______________

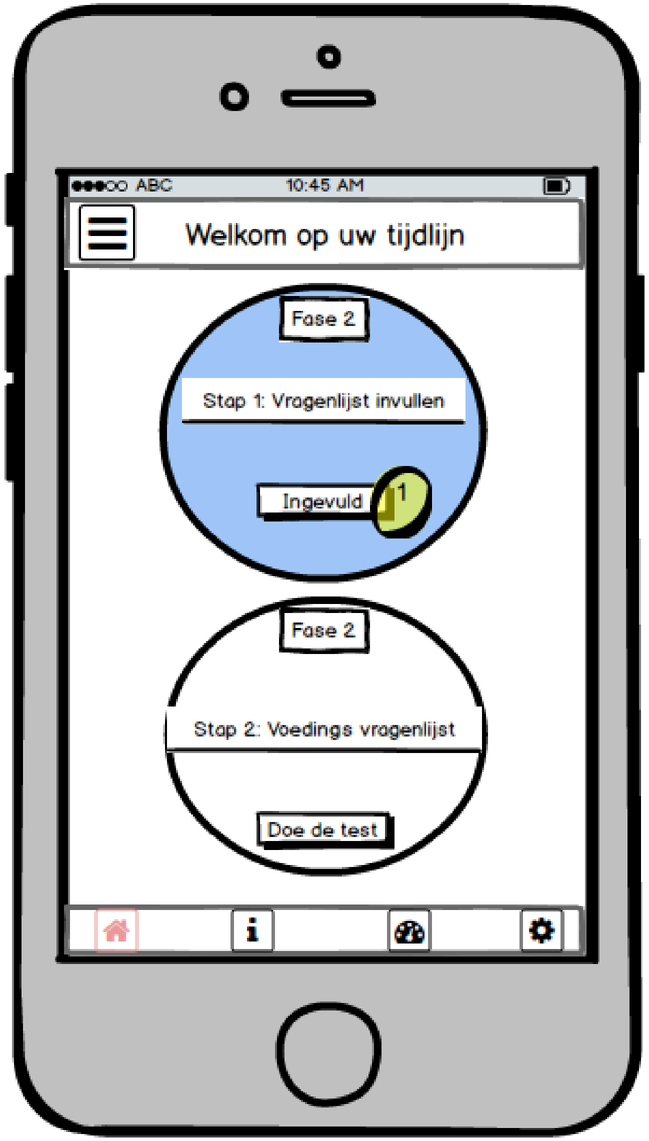

3.9 Informatie

Omschrijving:
Alle informatie die relevant is voor de gebruiker wordt hier gebundeld. Via de bottom toolbar kan deze versneld worden geraadpleegd.

Onderdelen:
1. De informatie pagina heeft een zoekfunctie. Alle onderdelen met corresponderende teksten worden als zoekresultaat getoond.
2. De elementen van het informatie menu worden hier genoemd. Door op een onderdeel te klikken wordt de bijbehorende tekst geopend.
3. In de bottom toolbar is door de afwijkende kleur is te zien dat dit menu momenteel geopend is.

Opmerkingen:

____

____

____

Paraaf: ____________

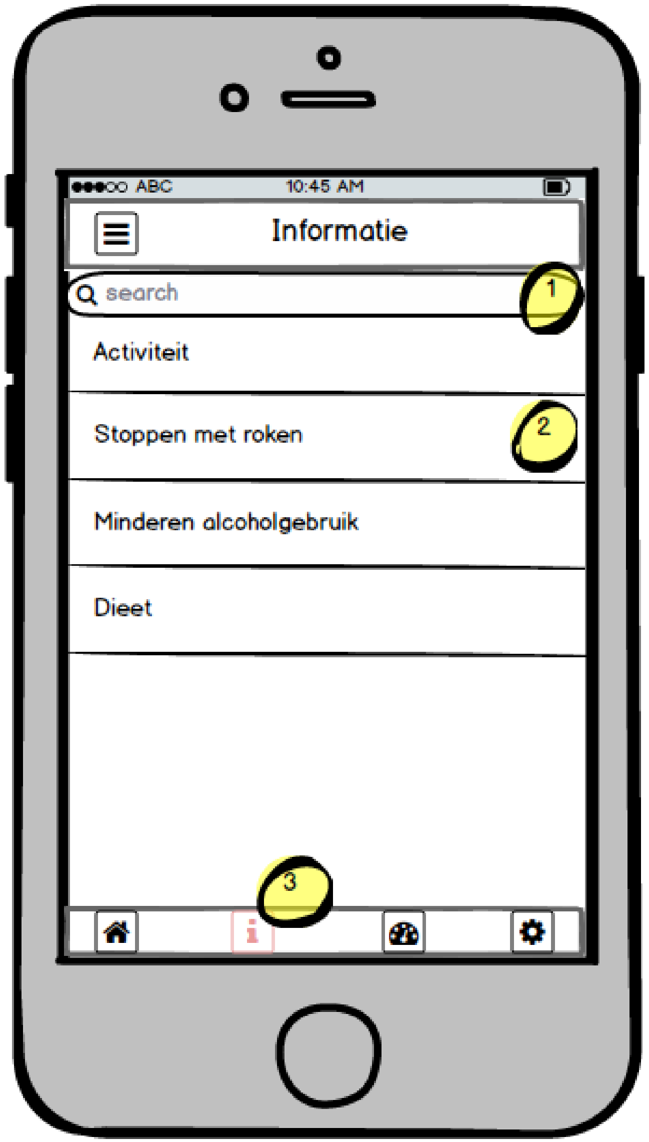

3.9.1 Informatie – Activiteit

Omschrijving:
Als voorbeeld is hier de tekst van activiteit uitgewerkt.

Onderdelen:
1. Informatie kan bestaan uit tekst, afbeeldingen en/of tabellen. Hierbij wordt zoveel mogelijk reeds bestaand materiaal gebruikt. De controle en interventie groep krijgen hierdoor dezelfde oefeningen.
2. Per onderdeel wordt uitgelegd hoe een oefening moet worden uitgevoerd.
3. Als de volledige tekst niet op de pagina past, kan worden gescrold.

Opmerkingen:

____

____

____

Paraaf: _______________

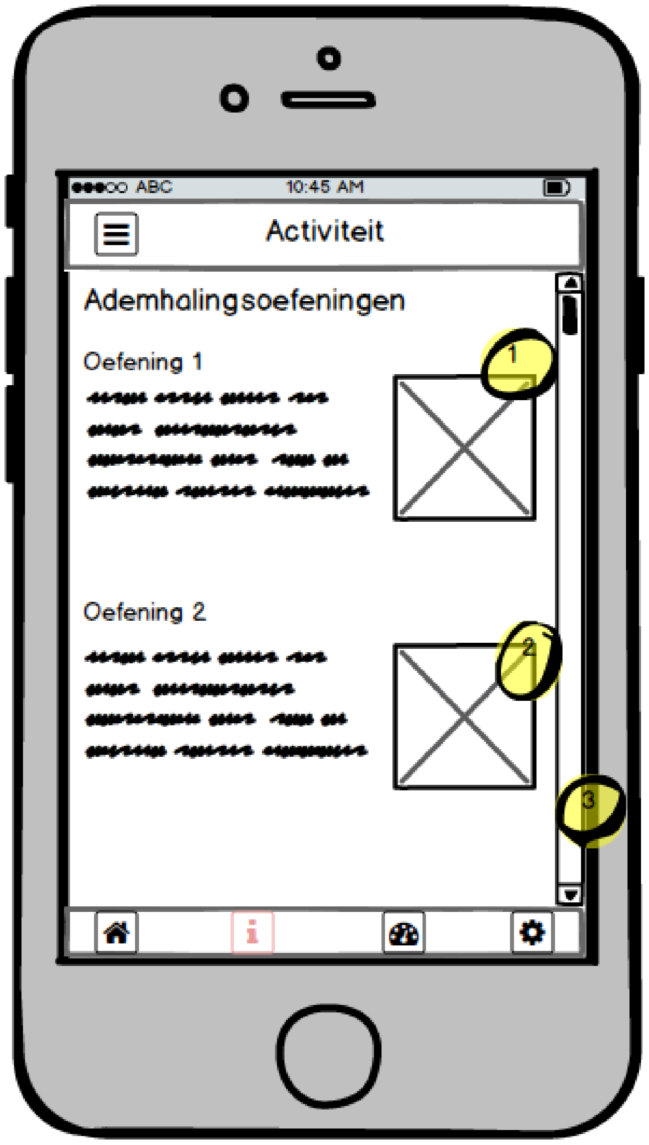

3.10 Dashboard

Omschrijving:
Op het dashboard wordt de voortgang van het herstel getoond, opgedeeld in verschillende onderdelen.
Door een onderdeel naar boven te slepen, wordt dit element van de voortgang in het groot getoond. De overige onderdelen worden als miniatuur getoond.
De gebruiker wordt gestimuleerd om alle cirkels gevuld te krijgen.

Onderdelen:

1. Op dit moment is “Activiteit” geselecteerd. Dagelijks heeft de gebruiker een te lopen aantal stappen als doel. De voortgang wat betreft dit doel wordt hier grafisch weergegeven.
2. Ook de minimale eisen voor ontslag waar aan voldaan moet worden, worden gevisualiseerd.
3. Net als de dagelijkse calorische intake.
4. In de bottom toolbar wordt met een afwijkende kleur aangegeven dat dit onderdeel momenteel geopend is.

Opmerkingen:

____

____

____

Paraaf: ______________

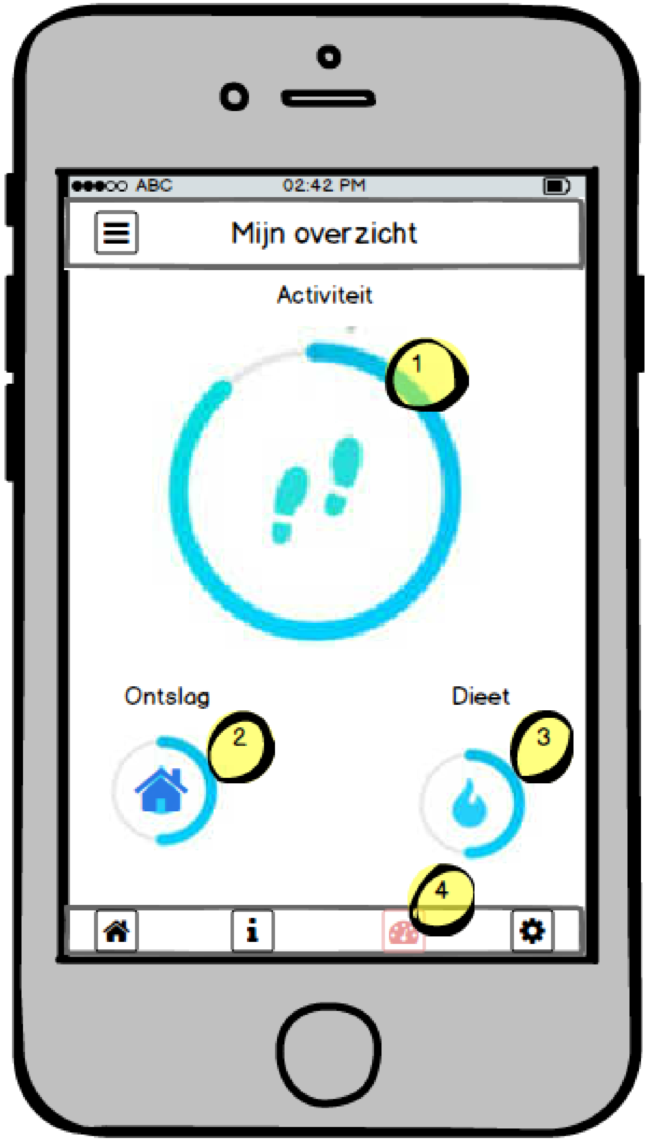

3.11 Instellingen

Omschrijving:
De instellingen voor meerdere onderdelen kunnen worden gewijzigd. De gebruiker komt daarom eerst in een overzichtsmenu.

Onderdelen:
1. Door hier te klikken, navigeert de gebruiker naar de operatie instellingen. Zie hiervoor 3.11.1.
2. Het informed consent “Uw toestemming”, is hier terug te vinden en in te trekken, zie 3.11.2.
3. Instellingen voor het gebruik van de app zijn via dit menu te allen tijde te wijzigen. Zie 3.11.3.
4. Via deze knop komt men op een pagina met informatie over de app. Zie 3.11.4.
5. In de bottom toolbar wordt met een afwijkende kleur aangegeven dat dit onderdeel momenteel geopend is.

Opmerkingen:

____

____

____

Paraaf: ______________

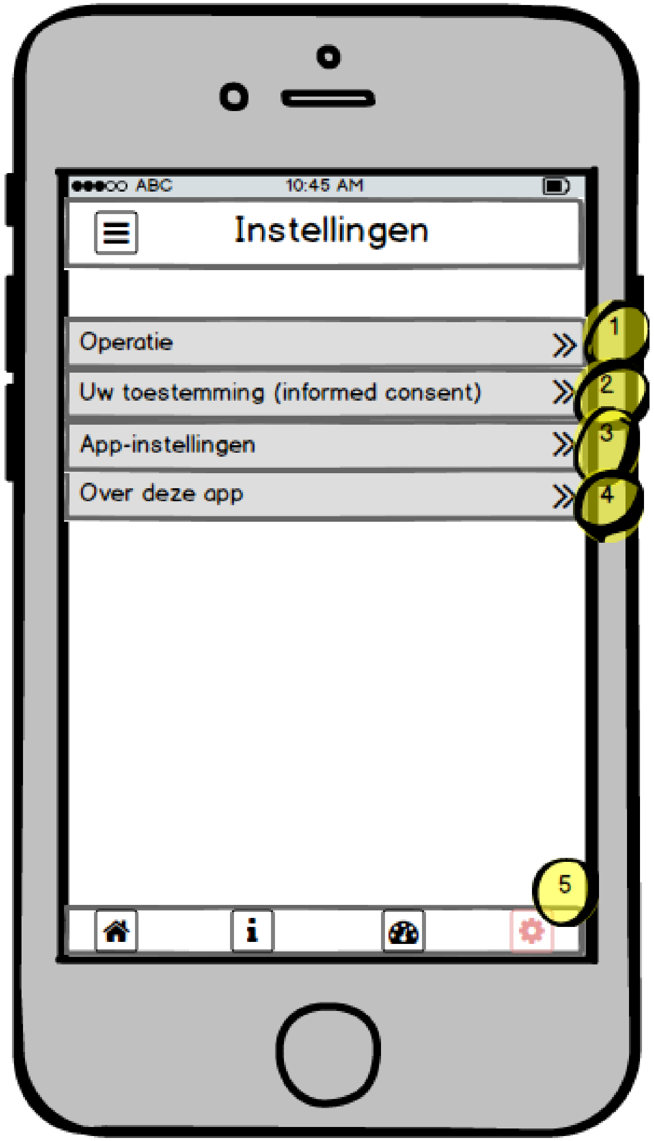

3.11.1 Instellingen - Operatie

Omschrijving:
Voor het genereren van de tijdlijn is het belangrijk dat het type operatie (zie bijlage 1) en de operatiedatum bekend zijn. Bij de eerste keer installeren wordt de gebruiker geholpen bij het starten van de app, een operatie datum kan echter verplaatst worden en peroperatief kan worden besloten een andere operatie uit te voeren. In dit menu kunnen de instellingen worden gewijzigd.

Onderdelen:
1. Hier kan het type operatie worden ingevoerd. Zie het scherm bij 3.11.1.1
2. Hier kan de operatiedatum worden gewijzigd. Zie het scherm bij 3.11.1.2

Opmerkingen:

____

____

____

Paraaf: ____________

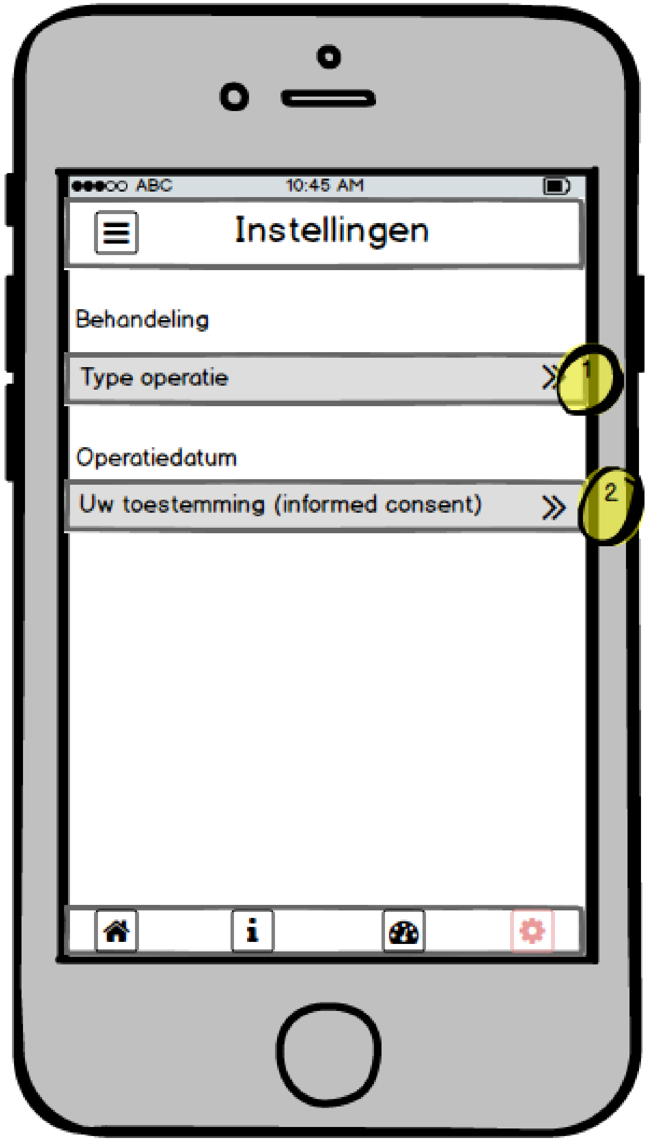

3.11.1.1 Instellingen – Operatie

Omschrijving:
In dit scherm kan de gebruiker het type operatie dat hij of zij zal moeten ondergaan selecteren.

Onderdelen:
1. De antwoordopties worden weergegeven in bijlage 1, screencontent instellingen.
2. Via deze knop keert de gebruiker terug in het instellingen-menu
3. Met deze knop kan een keuze worden bevestigd of een wijziging worden opgeslagen.

Opmerkingen:

____

____

____

Paraaf: ___________

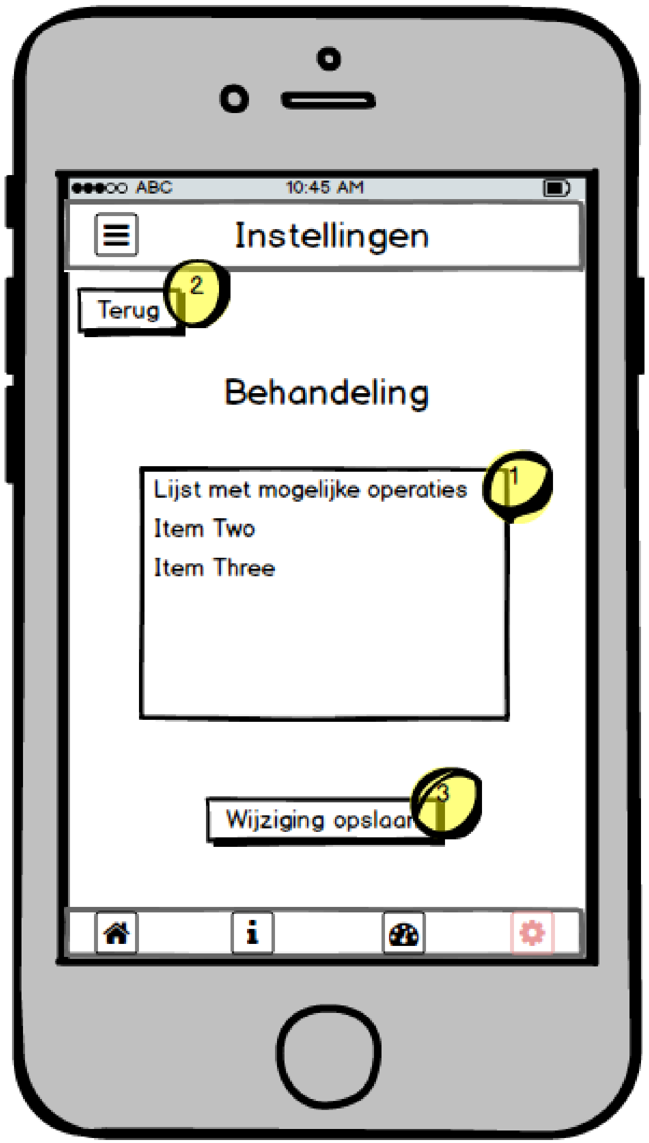

3.11.1.2 Instellingen – Operatie

Omschrijving:
De tijdlijn wordt gegenereerd op basis van de operatiedatum. De afwijking van de operatiedatum triggert notificaties en biedt de informatie die op dat moment van toepassing is aan binnen de app.

Onderdelen:

1. In de kalender kan de datum van operatie worden aangeklikt, als een datum is geselecteerd is dat zichtbaar doordat er een gekleurde cirkel omheen zichtbaar wordt.
2. Via deze knop keert de gebruiker terug naar het instellingen-menu
3. Met deze knop kan een keuze worden bevestigd of een wijziging worden opgeslagen.

Opmerkingen:

___

___

___

Paraaf: ___________

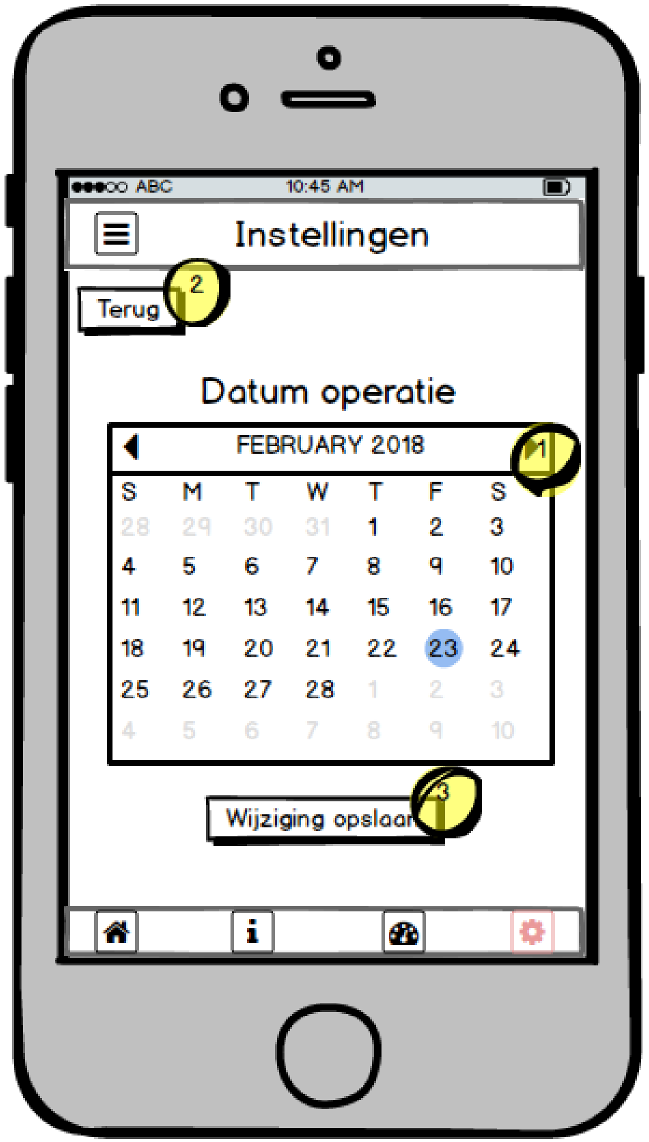

3.11.2 Informed Consent

Omschrijving:
Zoals bij elk wetenschappelijk onderzoek, hebben deelnemers de mogelijkheid op elk moment van verdere deelname af te zien. Dat kan in de app (maar ook op papier) worden aangegeven.

Onderdelen:

1. Hier staat uitgelegd waar mensen hier hun toestemming voor intrekken.
2. Als gebruikers deze check-box aanvinken doen zij niet langer mee aan de studie, maar hun eerder verzamelde gegevens mogen bewaard blijven.
3. Als gebruikers deze check-box aanvinken worden ook hun eerder verzamelde gegevens uit het onderzoek verwijderd.
4. Met deze knop kan de keuze worden bevestigd. De pop-up van 3.11.2.1 wordt getoond.

Opmerkingen:

____

____

____

Paraaf: ___________

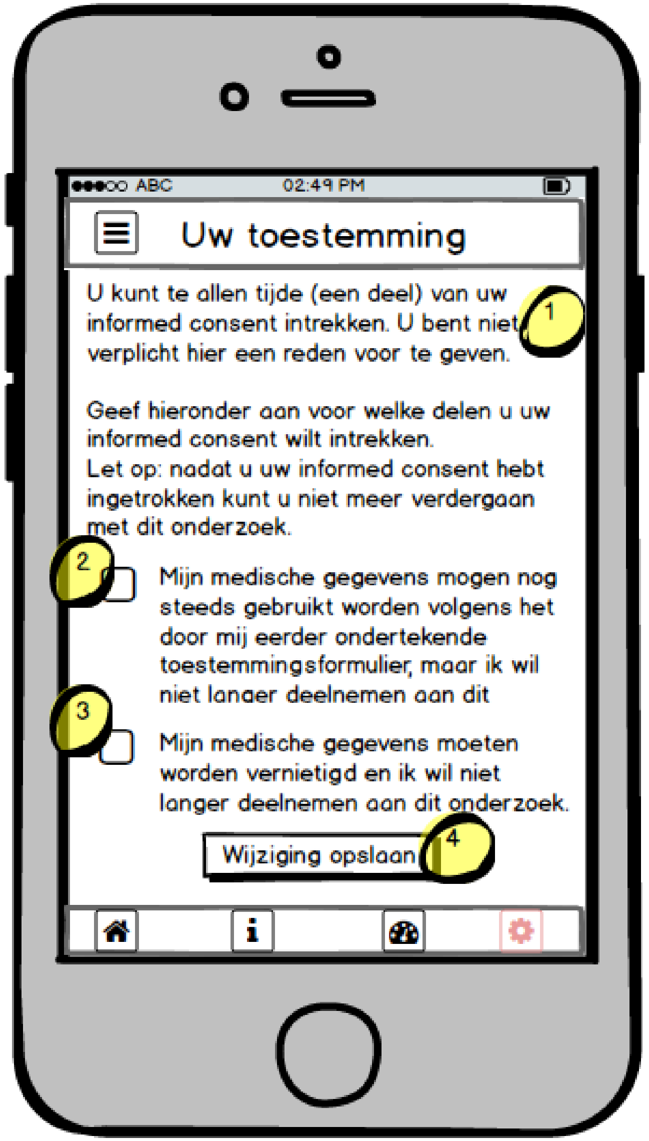

3.11.2.1 Pop-up

Omschrijving:
Als een gebruiker in het scherm 3.11.2 op wijziging opslaan klikt wordt deze pop-up geopend. Als op nee wordt geklikt worden wijzigingen niet doorgestuurd.

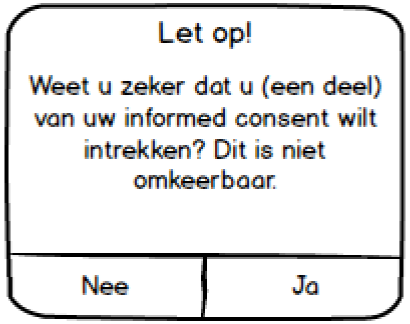

Opmerkingen:

____

____

____

Paraaf: ___________

3.11.3 App-instellingen

Omschrijving:
De voorkeuren voor het gebruik van de app kunnen te allen tijden via dit menu worden gewijzigd.

Onderdelen:

1. Hier kan de gebruiker het wachtwoord wijzigen, zie ook 3.11.3.1
2. De gebruiker kan ook voorkeuren aangeven voor versneld inloggen, zie paragraaf 3.11.3.2.
3. De gebruiker geeft bij het downloaden van de app al dan niet toestemming voor het delen van gebruiksgegevens met Google Analytics. Deze keuze kan hier worden gewijzigd.
4. Via deze info-button kan aanvullende informatie over Google Analytics geopend worden. Zie paragraaf 3.11.3.4.
5. Met deze knop kan de keuze worden bevestigd.

Opmerkingen:

____

____

____

Paraaf: __________

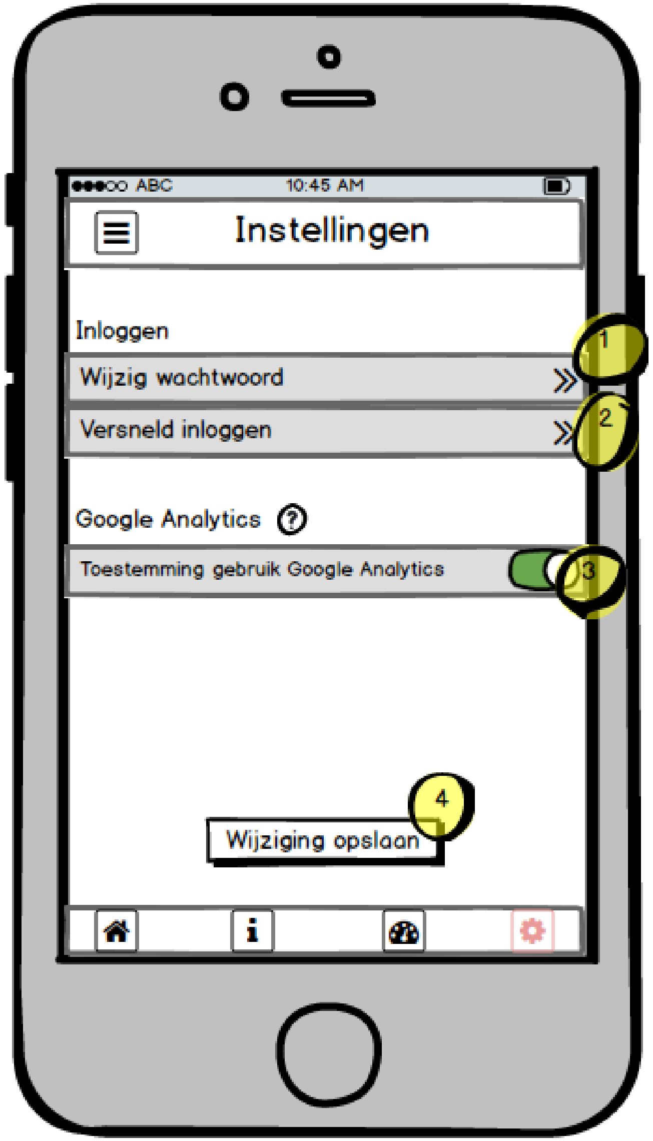

3.11.3.1 Wijzig wachtwoord

Omschrijving:
Voor de grootst mogelijke beveiliging van gegevens, wordt over het algemeen aangeraden een wachtwoord met enige regelmaat te wijzigen.

Onderdelen:
1. Hier wordt het nieuwe wachtwoord ingegeven.
2. Hier moet het wachtwoord herhaald worden om problemen door bijv. spelfouten te voorkomen.
3. Met deze knop kan de keuze worden bevestigd.

Opmerkingen:

____

____

____

Paraaf: ___________

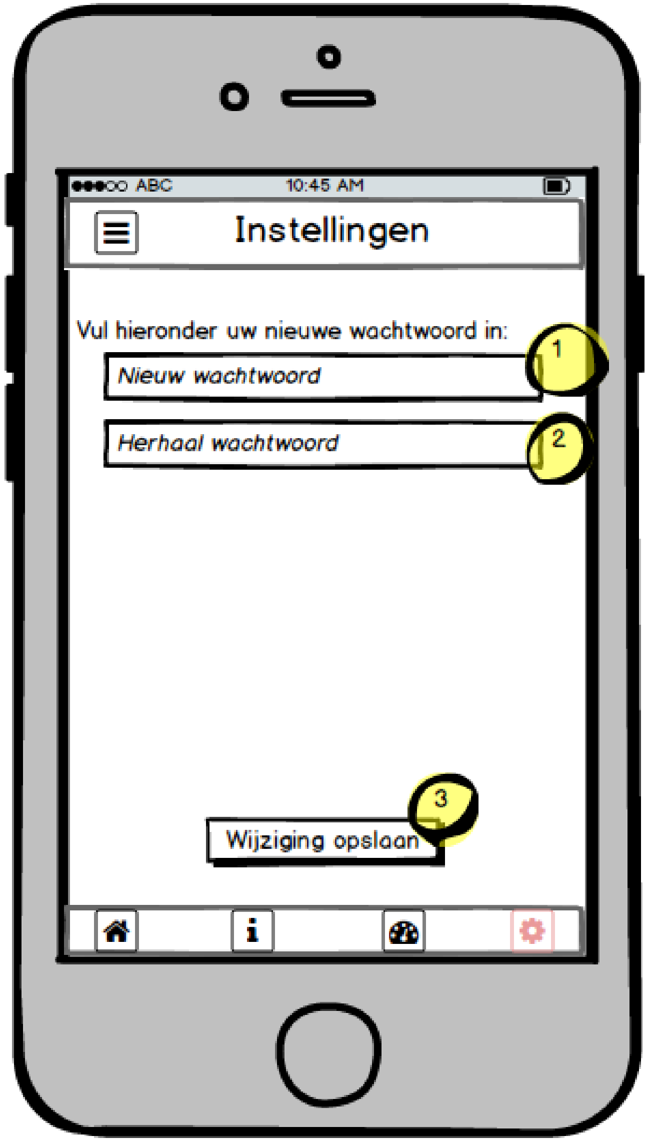

3.11.3.2 Versneld inloggen

Omschrijving:
Bij elk gebruik moet de app opnieuw ontgrendeld worden, dit om gegevens die wél op de app opgeslagen worden, zo goed mogelijk te beschermen. Om de gebruiksvriendelijkheid te vergroten worden opties geboden die een snellere inlog mogelijk maken.

Onderdelen:
1. Gebruikers kunnen een viercijferige pincode instellen. Daarna wordt de pop-up van pagina 3.11.3.1 getoond.
2. Smartphones bevatten allerlei sensoren, hiermee wordt toestemming gegeven om met de vinger-afdruk de app te ontgrendelen.
3. De laatste generatie smartphones kan ook op basis van gezichtsherkenning apps ontgrendelen. Hiermee kan toestemming worden gegeven gezichtsherkenning ook te gebruiken voor het ontgrendelen van deze app.
4. Met deze knop kan de keuze of een eventuele wijziging worden bevestigd.

Opmerkingen:

____

____

____

Paraaf: ___________

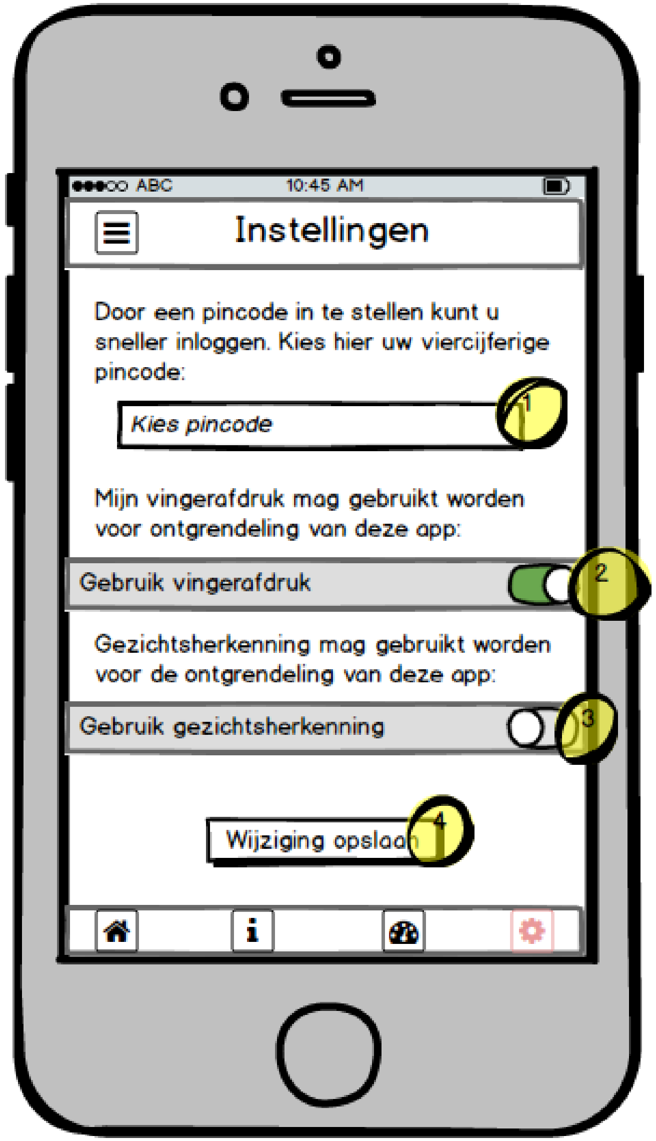

3.11.3.3 Pop-up pincode

Omschrijving:
Na het kiezen van een pincode wordt deze bevestiging getoond.

Opmerkingen:

____

____

____

Paraaf: ___________

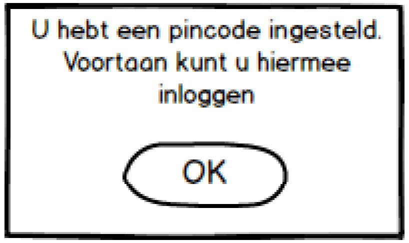

3.11.3.4 Info-button Google Analytics

Omschrijving:
Hier wordt aanvullende informatie over Google Analytics gegeven.

Opmerkingen:

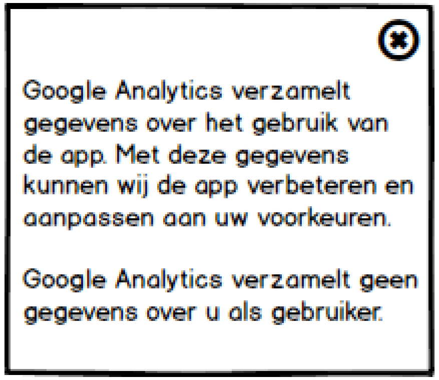

Paraaf: ___________

3.11.4 Over deze app

Omschrijving:
Hier zijn informatie over de app en de disclaimer terug te vinden.

Onderdelen:
1. Over deze app is op dit moment ingeklapt. Door op de pijl te klikken klapt het menu uit. Voor de tekst zie bijlage 2.
2. De disclaimer is op dit moment uitgeklapt. Door nogmaals op de pijl te klikken wordt de disclaimer weer ingeklapt.
3. Hier is de volledige tekst te lezen. De volledige tekst wordt momenteel gereviseerd en zal separaat aangeleverd worden.
4. Als de tekst niet volledig op de pagina past kun je er doorheen scrollen.

Opmerkingen:

____

____

____

Paraaf: ____________

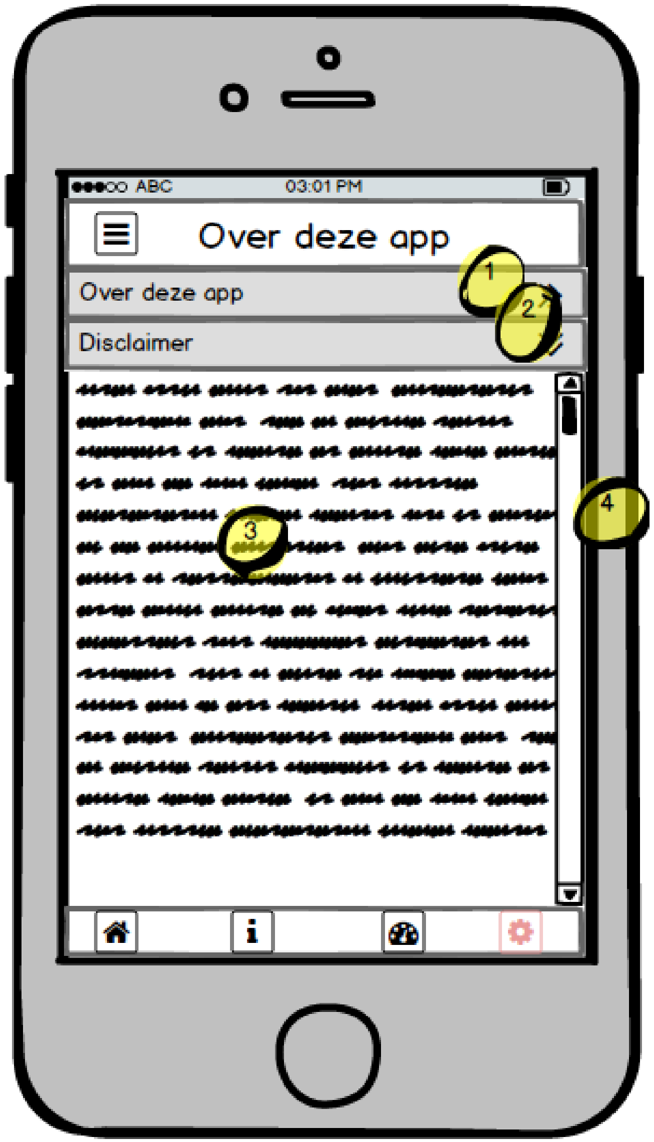

4.0 Handtekening voor akkoord

Indien u kleine wijzigingen wilt doorvoeren, dan willen we u vragen deze te noteren in het “Opmerkingen” deel. Mocht u grote wijzigingen willen doorvoeren, dan verzoeken we u contact op te nemen via e-mail of telefoon: marilou@everywhereIM.com/ 035-7370521
Indien u akkoord bent met dit functioneel ontwerp, verzoeken wij u de volgende stappen te volgen:

1. Print het functioneel ontwerp
2. Parafeer alle schermen
3. Plaats de datum, plaats, naam, functie en uw handtekening op deze pagina
4. Scan het hele document en mail het geheel naar marilou@everywhereIM.com

everywhereIM
t.a.v. Marilou Jansen
Paasheuvelweg 25, Wing 5D
1105 BP Amsterdam

Door dit functioneel ontwerp te ondertekenen geef ik – als opdrachtgever – aan akkoord te gaan met de functionaliteiten zoals deze staan omschreven in dit functioneel ontwerp. Ik ben me bewust van het feit dat wijzigingen die in een later stadium doorgevoerd moeten worden en die impact hebben op de functionaliteit van de app zullen worden behandeld als een change request.

Plaats: ________________________ Naam: ________________________

Datum: ________________________ Functie: ________________________

Handtekening: ________________________

5.0 Disclaimer

everywhereIM heeft dit document met grote zorg samengesteld. Zij garandeert echter niet de juistheid en de volledigheid van de inhoud van dit document. De inhoud van dit document, waaronder begrepen informatie en de teksten mag niet worden verveelvoudigd, opgeslagen in een gegevensbestand, of openbaar gemaakt worden, in enige vorm of op enige wijze dan ook, elektronisch, door fotokopieën, elektronisch, opnamen, of welke manier, dan ook, zonder voorafgaande schriftelijke toestemming van everywhereIM.

everywhereIM is in geen geval aansprakelijk voor enige directe, incidentele, indirecte gevolgschade, inclusief maar niet beperkt tot verlies van opbrengst of winst, bedrijfsschade, of schade die voortkomt uit of in verband staat met het gebruik of de onmogelijkheid van gebruik van dit document. U erkent dat dit document “as is” geleverd wordt zonder uitdrukkelijke of impliciete garantie, en, voor zover de van toepassing zijnde wet het mogelijk maakt, geeft everywhereIM geen enkele uitdrukkelijke of impliciete verklaring of garantie met inbegrip van maar niet beperkt tot de garantie van verhandelbaarheid of geschiktheid voor een bepaald doel.

Wijziging disclaimer
everywhereIM behoudt zich het recht voor om de disclaimer op ieder gewenst moment, zonder voorafgaande melding hiervan, te wijzigen.

Bijlage 1

Screencontent Instellingen

-Dikke darm operatie ivm (mogelijk) kwaadaardige aandoening
-Dikke darm operatie ivm (mogelijk) kwaadaardige aandoening + in opzet tijdelijk stoma
-Dikke darm operatie ivm (mogelijk) kwaadaardige aandoening + in opzet blijvend stoma
-Dikke darm operatie ivm goedaardige aandoening
-Dikke darm operatie ivm goedaardige aandoening + in opzet tijdelijk stoma
-Dikke darm operatie ivm goedaardige aandoening + in opzet blijvend stoma
-Aanleggen stoma

Bijlage 2

Versienummer: x.x.x.

Hoewel bij het opstellen van deze applicatie uiterste zorgvuldigheid is betracht, kan everywhereIM, noch de deelnemende ziekenhuizen, geen enkele aansprakelijkheid aanvaarden voor eventuele onjuistheden, genomen beslissingen gebaseerd op de inhoud van deze applicatie, noch voor enige schade, overlast of ongemak die voortvloeien uit of samenhangen met het gebruik van de inhoud van deze applicatie.
Deze applicatie biedt enkel reeds in eerder onderzoek bewezen informatie en is daarom geen medisch hulpmiddel en derhalve niet CE-gemarkeerd.
